# Nitric oxide regulates the firing rate of neuronal subtypes in the guinea pig ventral cochlear nucleus

**DOI:** 10.1111/ejn.14572

**Published:** 2019-10-10

**Authors:** Adam Hockley, Joel I. Berger, Paul A. Smith, Alan R. Palmer, Mark N. Wallace

**Affiliations:** ^1^ Medical Research Council Institute of Hearing Research School of Medicine University of Nottingham Nottingham UK; ^2^ School of Life Sciences University of Nottingham Nottingham UK; ^3^ Department of Otolaryngology Kresge Hearing Research Institute University of Michigan Ann Arbor MI USA; ^4^ Department of Neurosurgery University of Iowa Iowa City IA USA; ^5^ Hearing Sciences School of Medicine University of Nottingham Nottingham UK

**Keywords:** auditory system, central gain, neuromodulation, nitric oxide synthase

## Abstract

The gaseous free radical, nitric oxide (NO) acts as a ubiquitous neuromodulator, contributing to synaptic plasticity in a complex way that can involve either long term potentiation or depression. It is produced by neuronal nitric oxide synthase (nNOS) which is presynaptically expressed and also located postsynaptically in the membrane and cytoplasm of a subpopulation of each major neuronal type in the ventral cochlear nucleus (VCN). We have used iontophoresis in vivo to study the effect of the NOS inhibitor L‐NAME (L‐NG‐Nitroarginine methyl ester) and the NO donors SIN‐1 (3‐Morpholinosydnonimine hydrochloride) and SNOG (S‐Nitrosoglutathione) on VCN units under urethane anaesthesia. Collectively, both donors produced increases and decreases in driven and spontaneous firing rates of some neurones. Inhibition of endogenous NO production with L‐NAME evoked a consistent increase in driven firing rates in 18% of units without much effect on spontaneous rate. This reduction of gain produced by endogenous NO was mirrored when studying the effect of L‐NAME on NMDA(*N*‐Methyl‐D‐aspartic acid)‐evoked excitation, with 30% of units showing enhanced NMDA‐evoked excitation during L‐NAME application (reduced NO levels). Approximately 25% of neurones contain nNOS and the NO produced can modulate the firing rate of the main principal cells: medium stellates (choppers), large stellates (onset responses) and bushy cells (primary‐like responses). The main endogenous role of NO seems to be to partly suppress driven firing rates associated with NMDA channel activity but there is scope for it to increase neural gain if there were a pathological increase in its production following hearing loss.

## Graphical abstract

A nitric oxide (NO) donor or synthesis blocker was applied iontophoretically to physiologically identified classes of neurones in the ventral cochlear nucleus. Approximately a quarter of neurones responded: the main effect of endogenous NO was a suppression of driven activity. NO synthase levels are raised following noise exposure, suggesting that NO may contribute to a compensatory control of firing rates.

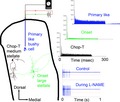

**Abbreviations**
CF: characteristic frequency
cGMP: cyclic guanosine monophosphate
Ch: chopper
eNOS: endothelial nitric oxide synthase
FSL: first spike latency
gr: granule cells
ISI: inter‐spike interval
L‐NAME: Nω‐Nitro‐L‐arginine methyl ester
NADPH: reduced nicotinamide adenine dinucleotide phosphate
NMDA: *N*‐Methyl‐D‐aspartic acid
nNOS: neuronal nitric oxide synthase
NO: nitric oxide
On: onset
Ph: phase‐locked
Pr: primary‐like
PSTH: peri‐stimulus time histogram
SIN‐1: 3‐Morpholinosydnonimine hydrochloride
un: unclassified
VCN: ventral cochlear nucleus

## 1 INTRODUCTION

It has been suggested that homoeostatic mechanisms maintain firing rates throughout the auditory nervous system, such that, for example, the output from the ventral cochlear nucleus (VCN) to the higher centres of the brain remains close to a long term average even when there are fluctuations in the peripheral input (Noreña, 2011; Schaette & McAlpine, 2011). However, the details of the neural mechanisms involved are unknown (Auerbach et al., 2014).

Homoeostatic plasticity is mediated by neuromodulators such as nitric oxide (NO) which facilitate or suppress the strength of synaptic transmission (Steinert et al., 2011). The NO‐forming enzyme, NOS has three main isoforms: an inducible form which is present in reactive glial cells, but which is not present in the control, healthy VCN (Coomber, Kowalkowski, Berger, Palmer, & Wallace, 2015) and two calcium‐dependent isoforms that are located in either endothelial cells (eNOS) or some neurones (nNOS). The endothelial eNOS is mainly involved in controlling vascular tone, but may provide a uniform background level of NO that determines the baseline levels of nearby neurones (Garthwaite, 2008). The nNOS has been identified in many stages of the auditory system, from the cochlea through the cochlear nucleus, superior olivary complex (SOC) and inferior colliculus to the auditory cortex (Baizer et al., 2014; Burette, Petrusz, Schmidt, & Weinberg, 2001; Coote & Rees, 2008; Eliasson, Blackshaw, Schell, & Snyder, 1997; Fessenden, Altschuler, Seasholtz, & Schacht, 1999; Fessenden, Coling, & Schacht,^1994^; Lee, Cho, Huh, Cha, & Yeo, 2008; Rodrigo et al., 1994). The presence of nNOS throughout the auditory system suggests that NO contributes significantly to auditory processing and that it does so in a number of ways. NO has been shown to modulate postsynaptic excitability at the medial nucleus of the trapezoid body (MNTB; Steinert et al., 2008, 2011), to reduce the activity of the neuronal potassium–chloride cotransporter in the superior paraolivary nucleus (Yassin et al., 2014) and to affect the efficiency of I_H_ currents in the SOC nuclei (Kopp‐Scheinpflug, Pigott, & Forsythe, 2015). NO also has a role in the NMDA‐dependant potentiation of synapses in a polysynaptic network of chopper cells (T‐stellate) in the VCN (Cao, Lin, Sugden, Connors, & Oertel, 2019).

nNOS is present both in the spiral ganglion neurones that provide the afferent innervation to the VCN (Zdanski et al., 1994) and in the VCN projection neurones. These are the globular and spherical bushy cells, which show primary‐like peri‐stimulus time histograms (PSTHs; Smith & Rhode, 1987); small‐ to medium‐sized stellate cells, with chopper PSTHs (Palmer, Wallace, Arnott, & Shackleton, 2003; Smith & Rhode, 1989); and large stellate cells and octopus cells (OC) with distinctive onset responses (Arnott, Wallace, Shackleton, & Palmer, 2004; Oertel, Wu, Garb, & Dizack, 1990; Rhode, Oertel, & Smith, 1983). The afferent inputs from the auditory nerve and the majority of VCN neurones with intrinsic branches are excitatory, while the large stellate onset cells and some small chopping stellates are glycinergic (Doucet, Ross, Gillespie, & Ryugo, 1999; Ngodup & Trussell, 2019). All of these intrinsic neurones can contain nNOS, but within each class there are large variations in the quantity of enzyme present (Coomber et al., 2015). Recent work in the inferior colliculus has indicated that nNOS may either be distributed throughout the cytoplasm or have a punctate distribution on the surface of neurones and be associated with NMDA receptors at the postsynaptic density (Olthof‐Bakker, Gartside, & Rees, 2019). This may also be true in the VCN.

Various manipulations of peripheral afferent input have been used to study NO in the auditory system. Cochleotomy, resulting in hugely decreased input, produces decreased spontaneous firing rates (Koerber, Pfeiffer, Warr, & Kiang, 1966) and increased nNOS expression (Chen, Huang, Wang, & Chen, 2004) in the VCN, primarily by a redistribution of nNOS from being mostly in the cytoplasm to being mostly associated with NMDA receptors in the membrane. Other less traumatic peripheral alterations such as acoustic over exposure (Alvarado et al., 2016; Coomber et al., 2014) also result in increases to cochlear nucleus nNOS expression. Following noise exposure, changes in nNOS are correlated with behavioural evidence of tinnitus (Coomber et al., 2014). As NO can alter synaptic plasticity, it may have a role in the gain changes that have been suggested as a potential mechanism underlying tinnitus (Noreña, 2011).

Such data have led to the postulate that NO levels in the VCN are involved in homoeostatic plasticity (Coomber et al., 2015). To test this hypothesis, we have used the iontophoretic application of a NOS inhibitor and two NO donors, while recording from identified single units in vivo. For the inhibitor we used L‐NAME which is a water‐soluble, non‐selective inhibitor that is suitable for iontophoresis (Carletti, Ferraro, Rizzo, Friscia, & Sardo, 2012). Initially, we used SIN‐1 as an NO donor as it has often been used successfully for in vivo studies in the past (Galati et al., 2008). However, like NOS itself, SIN‐1 is not a pure source of NO as its decomposition produces equal amounts of NO and the superoxide ion that combines to form peroxynitrite (Feelisch, Ostrowski, & Noack, 1989). The peroxynitrite can then either oxidize sensitive cellular components or lead to an active intermediate by reacting with glutathione to produce SNOG which can then release NO to activate guanylyl cyclase (Schrammel, Pfeiffer, Schmidt, Koesling, & Mayer, 1998). Thus we also used the iontophoresis of SNOG directly as it has been shown to be an effective source of NO when iontophoresed onto neurones in other parts of the brain (Carletti et al., 2012).

If NO is specifically involved in modulating neuronal firing rates, then both the inhibitor and donors should be effective, and their effects may vary depending on whether or not the neurone is acoustically driven. Much of our information about NO action has derived from slice preparations which involved electrical stimulation and which typically were allowed to equilibrate for up to an hour. As Steinert et al. (2011) demonstrate, much of the endogenous nitrergic action may be lost in this hour. Here we used an intact in vivo preparation that has allowed precisely timed, physiological sensory stimulation (acoustic tones presented to the ear) combined with extremely local application of NO donors and an inhibitor (by iontophoresis directly onto the target cell) and the ability to define (by physiological profile at least) the cell type under study. Thus, the data we present here should represent a valid demonstration of the type of effects that result from nitrergic action during mild stimulation.

## 2 MATERIALS AND METHODS

All procedures were carried out under authority of the UK Home Office under the Animals (Scientific Procedures) Act 1986. Experiments were run in accordance with the European Communities Council Directive 1986 (86/609/EEC) and the approval of the Animal Welfare and Ethical Review Body at the University of Nottingham, UK.

### 2.1 Animals

Male and female tricolour guinea pigs weighing 400–1,200 g were used. For each experimental procedure applied to a group, we attempted to use roughly equal numbers of animals of each sex. However, as we were using animals from our own small colony and did not want to cull animals, there was sometimes an imbalance in the numbers of each sex. Guinea pigs were group‐housed in floor pens or large, well‐ventilated cages with environmental enrichment (cardboard tubes, plastic houses and wooden gnawing blocks) on a 12/12 hr light‐dark cycle, with food and water readily available.

### 2.2 Histology

Stained sections showing nNOS were available from our previous study of the effect of noise exposure on NOS expression in the VCN (Coomber et al., 2015). Sections were cut at 50 μm and endogenous peroxidase removed by immersing in phosphate buffer (100 mM, pH 7.4) containing 0.3% H_2_O_2_ and 10% methanol, followed by phosphate buffer containing 5% normal horse serum (NHS) and 0.5% Triton X‐100. Sections were then incubated with monoclonal anti‐nNOS primary antibody (1:2,000; N2280, Sigma) in buffer containing 5% NHS and 0.5% Triton X‐100 overnight at 4°C. Subsequently, sections were washed (3 × 10 min in buffer), before incubation with biotinylated anti‐mouse secondary antibody (1:100; Vector Laboratories, UK) for 2 hr at room temperature. Sections were then washed and incubated in buffer containing Vectastain ABC *Elite* solution (Vector Laboratories) and 1% NHS. After a further series of washes, the sections were placed in 0.05% diaminobenzidine tetrahydrochloride (DAB) solution, in buffer, for 10 min, followed by a solution of 0.001% hydrogen peroxide in DAB solution for an additional 10 min. The sections were then washed in buffer to halt the DAB reaction, mounted on coated slides, air‐dried, dehydrated and cover‐slipped. These were re‐examined to study the distribution of nNOS staining and determine if it was associated with postsynaptic densities in the same way as for the inferior colliculus (Olthof‐Bakker et al., 2019). We also separately stained 50 μm sections for Nissl substance with a standard cresyl violet stain (0.5% dissolved in 0.2 M acetate buffer at pH 3.9) to allow comparison of the density of neurones in the VCN. In addition, we compared the nNOS stained VCN sections with those loaded with chelated thallium ions to indicate the levels of potassium uptake and associated neuronal activity (Goldschmidt et al., 2010). In two control animals, the external jugular vein was cannulated under deep urethane anaesthesia. A freshly prepared solution of thallium chelated with diethyldithiocarbamate consisting of 3 ml of a 0.2% suspension was injected into the vein over a period of 3 min and flushed through with physiological saline. The animals were then given a lethal dose of pentobarbitone (i.v.) 2 min later and the brains prepared for autometallography as described previously (Coomber et al., 2011). Coronal sections (50 μm) were cut through the VCN and processed to demonstrate the presence of thallium ions (Goldschmidt et al., 2010).

### 2.3 Surgery

Guinea pigs were anaesthetized with 20% w/v urethane (4.5 ml/kg i.p.; Sigma) and Hypnorm solution (fentanyl/fluanisone; 0.2 ml i.m.). Further injections of Hypnorm of up to 0.2 ml i.m. were administered when required to maintain surgical anaesthesia. Guinea pigs were artificially respirated with 100% oxygen (tidal volume 3.5 ml, respiratory rate 100 per minute) and the temperature was maintained at 38 ± 0.5°C. Guinea pigs were placed within a sound‐attenuating and electrically earthed chamber, positioned in a stereotaxic frame with hollow speculae lined up to the tympanic membrane. The skull was exposed and levelled between 5 and 13 mm anterior to ear bar zero (Rapisarda & Bacchelli, 1977). An insect pin was attached along the centre of the skull to mark the midline. The skull over the left cerebellum was removed by the use of an electric drill and rongeurs at between 1 and 5 mm lateral to the midline. Dura mater was excised and the exposed brain surface was kept moist by regular application of warm 0.9% saline or by a layer of agar (1.5% solution in 0.9% saline).

### 2.4 Sound presentation

Experiments were carried out in a sound‐attenuated chamber. Auditory stimuli were delivered monaurally via a closed‐field system (modified Radioshack 40‐1377 tweeters; M. Ravicz, Eaton Peabody Laboratory) coupled to damped 4‐mm‐diameter probe tubes, which fitted into the hollow earbars. The speakers were driven by a Tucker–Davis Technologies (TDT) System 3 (RZ2), controlled by Brainware (software developed by J. Schnupp, University of Oxford, UK). A probe tube microphone (Brűel and Kjaer 4134 with a calibrated 1‐mm probe tube) was used to calibrate the sound system close to the tympanic membrane. The sound system response was flat to within ±10 dB from 100 to 35,000 Hz (see Palmer & Shackleton, 2009 for an example calibration curve).

### 2.5 Iontophoresis

Extracellular drug application was achieved using five‐barrelled glass micropipettes (5B120F‐4; World Precision Instruments (WPI)). These were pulled with a microelectrode puller and the tips broken back to a width of 20 μm under a microscope using a glass ball. A taper‐to‐tip length of 10–15 mm was optimal to achieve the desired resistance and to reach the cochlear nucleus. Glass‐coated tungsten recording electrodes with a tip length of 10 μm were made in‐house (Bullock, Palmer, & Rees, 1988) and glued to the micropipette using 5‐min epoxy resin, with the tip extending 20–30 μm beyond the pipette barrels in order to minimize damage at the recording site.

The drug barrels of the 5‐barrelled glass pipette were filled with either Nω‐Nitro‐L‐arginine methyl ester hydrochloride (L‐NAME, 50 mM, pH 6.5), 3‐Morpholinosydnonimine hydrochloride (SIN‐1, 40 mM, pH 4.5), *N*‐Methyl‐D‐aspartic acid (NMDA, 10 mM, pH 8) and glutamate (100 mM, pH 8), all obtained from Sigma and dissolved in distilled water, or S‐Nitrosoglutathione (SNOG, 15 mM, pH 4.5 obtained from Abcam [ http://www.abcam.com]). Retaining currents of 10 nA were applied to drug barrels between ejections, with polarity opposite to the charge of the drug to be ejected. Retaining currents were positive for NMDA and glutamate and negative for L‐NAME, D‐NAME, SNOG and SIN‐1.

The central barrel was filled with 2 M NaCl solution, to allow current balancing, with the remaining barrels filled with drugs. Cycling was used to maintain drug concentrations at the electrode tip between recordings, where each drug barrel would be supplied with the ejection current for 8 s every 36 s. All currents were applied by a Neurophore (Digitimer) BH‐2 iontophoresis system, consisting of four IP‐2 iontophoretic pump modules and one BM‐2 control and balance module.

Each time a new electrode assembly was inserted into the brain, we checked that the assembly was working properly by recording from and stimulating a cerebellar neurone. Once a unit was isolated, ejection currents were applied to the glutamate barrel. An excitation response suggested that the iontophoresis set‐up was functioning correctly. The iontophoresis assembly was then advanced into the VCN and, after neuronal identification, experiments involving drug application begun.

### 2.6 Recordings

Electrodes were advanced through the cerebellum to the cochlear nucleus at a 45° angle using a Burleigh Inchworm stepping motor (Burleigh Instruments). The VCN was located stereotaxically, 3.5–4.5 mm lateral of midline, ±1 mm anterior/posterior of ear bar zero and 0–2 mm dorsal of ear bar zero.

The single‐channel recording electrode was connected through an HS‐2 headstage (×1 L; Axon Instruments Inc.) and an Axoprobe‐1A microelectrode amplifier (×100; Axon Instruments Inc.) then further amplified ×100 (IHR 4 Channel Amplifier). The resulting signal then passed to a TDT System 3, RZ2 processor for filtering (0.3–3 kHz), and online data collection was facilitated by Brainware at a sampling rate of 25 kHz. Spikes were detected online in Brainware by an arbitrarily set threshold, never less than double the peak‐to‐peak amplitude of the background noise to keep the risk of false positives at less than 5%.

### 2.7 Analyses

Upon isolation of a single unit based on spike height/waveform, the sound frequency at the minimum threshold was determined (characteristic frequency: CF) to confirm that the new unit had a CF that was different by at least one octave from the CF of the previous unit in the track, where drugs had been applied. Units were only analysed if they were at least 300 μm from a previous unit in the same track to minimize the risk of any previously applied drugs still having a residual effect. Single units were classified into one of five subgroups: primary‐like (Pr), chopper (Ch), phase‐locked (Ph), onset (On) or unclassified (Un) based on PSTHs to 50‐ms CF tones at 20 dB suprathreshold. Primary‐like units have temporal discharge patterns like those of primary auditory nerve fibres showing a stochastic discharge, which is maximal at onset and then adapts to a steady state: chopper units show preferred firing times resulting in regularly occurring spikes and peaks in their PSTH: Onset units show one or two peaks at the onset of their PSTH with low sustained firing rates thereafter. Phase locking occurs in response to low‐frequency sinusoids and in low‐CF units often obscures the shape of the PSTH preventing definite allocation to primary‐like, chopper or onset types (Arnott et al., 2004; Palmer et al., 2003; Rhode et al., 1983; Smith & Rhode, 1989).

Hour‐long, pure‐tone pulse trains were then presented at the characteristic frequency (200 ms tone pip, 800 ms silence, 3,600 repeats). Spike timings throughout the sweep were recorded, allowing offline analysis of the auditory‐driven firing rate (initial 200 ms) or spontaneous firing rate (final 700 ms). Spike timings that occurred within 100 ms following the offset of the tone pip were not included in analysis, in order to allow for poststimulus recovery of spontaneous activity.

### 2.8 Drug ejection periods and statistical analysis

**Figure 1:**
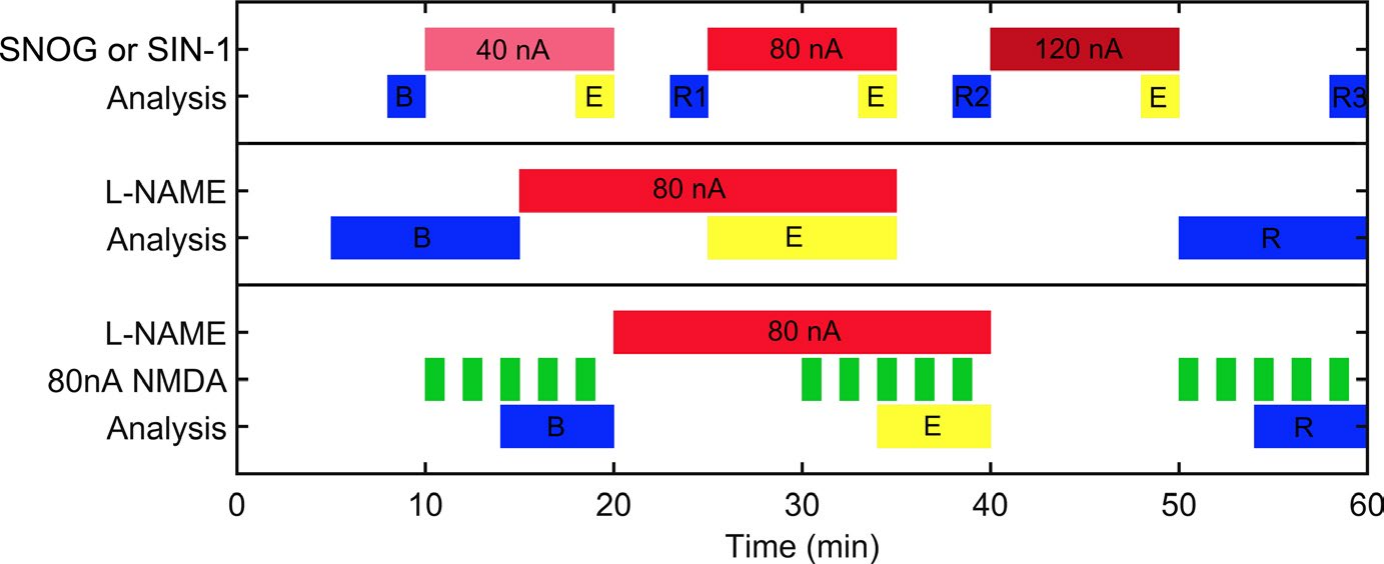
The timing of drug applications (red and green blocks) and response measurements. For statistical analysis in all iontophoresis experiments, yellow blocks labelled (E) show periods in which the response to the drugs were measured, blue blocks labelled (B) show baseline intervals and those labelled (R) show recovery intervals [Colour figure can be viewed at http://wileyonlinelibrary.com]

SIN‐1 and SNOG were applied for 10 min, three separate times over an hour, using increasing ejection currents of 40, 80 and 120 nA at 10–20, 25–35 and 40–50 min, respectively (Figure 1). Spike rates were computed in 10 s epochs at the following time points: the final 2 min of baseline prior to drug ejection (baseline: B), the final 2 min during each drug ejection (shown in yellow (E) in Figure 1) and the final 2 min of each recovery period (recovery: R1, R2 and R3). In chronological order, the seven analysis periods were: B, 40 nA, R1, 80 nA, R2, 120 nA and R3. Analysis periods were compared using a Kruskal–Wallis test with Dunn's multiple comparison test and responses were deemed significant if there was a significant change from R1—80 nA and R2—120 nA in the same direction, and a significant return towards baseline from 120 nA—R3.

L‐NAME was applied at 80 nA for 20 min (from 15 to 35 min) during an hour‐long recording (Figure 1). Pilot studies had shown that the build‐up of an effect of blocking nNOS activity had a slower time course than either of the NO donors. Spike rates were measured over 60 s epochs, and the final 10 min of drug ejection (shown yellow (E) in Figure 1) was compared with both the final 10 min of baseline (B) and the final 10 min of the recording (R) using a Kruskal–Wallis test with Dunn's multiple comparison test. If both comparisons showed significant changes in the same direction, the unit firing rate was deemed to have changed by a statistically significant degree.

The effect of L‐NAME on NMDA‐mediated excitation was measured during a 1‐hr tone pulse train. NMDA was applied at 80 nA for 1 min every 2 min between 10 and 20 min, 30 and 40 min and 50 and 60 min, while L‐NAME was applied at 80 nA from 20 to 40 min (Figure 1). This gave one baseline period of NMDA responses (B), one period of responses in the presence of L‐NAME (yellow (E) in Figure 1) and one recovery period (R). With these data in 10 s bins, the six bins with highest spike rate from the final three NMDA applications of each period were used. The baseline firing rate (mean of 180 s before the initial NMDA application of that period) was used to calculate the percentage change in firing rate evoked by NMDA. These increases for each period were compared using a Kruskal–Wallis test with Dunn's multiple comparison test.

### 2.9 Spike shape and phase‐locking analysis

The mean spike shape for each sweep (1 s) was calculated, from which the peak‐to‐peak amplitude and width at half amplitude were measured and plotted over the recording period. For statistical analysis, data were binned as before, over 1 min for L‐NAME and over 10 s for NO donors. Again, comparisons between analysis periods were made using a Kruskal–Wallis test with Dunn's multiple comparison test.

The strength and timing of phase locking in phase‐locked units was analysed for each analysis period by the vector strength method (Goldberg & Brown, 1969). The distribution of spikes in the period histogram (locked to a single period of the stimulus waveform) derived from each analysis interval was compared using a two‐sample Kolmogorov–Smirnov test. If phase locking during drug application was significantly different from baseline and recovery, and baseline and recovery were not significantly different, the phase locking of the unit was judged to have been altered.

## 3 RESULTS

### 3.1 nNOS histology

**Figure 2:**
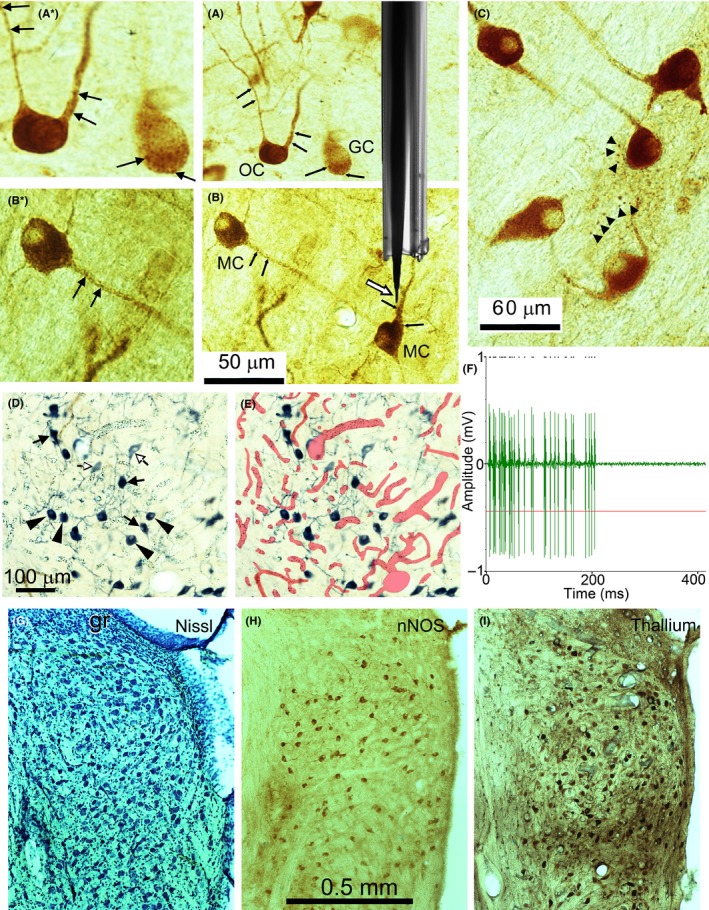
Local environment of neurones containing nNOS in the VCN. (a) The nNOS staining is associated with a number of structures and can produce dense cytoplasmic staining in the soma such as the octopus cell (OC) but it also forms distinct clumps that seem to be associated with the postsynaptic membrane of both somata and dendrites as indicated by black arrows on the octopus cell (OC) dendrites and globular bushy cell soma (GC). These cells are shown at higher power in panel a*. (b) The nNOS staining is also associated with discrete particles on the membrane of multipolar cells (MC, black arrows). Some of these particles are shown at higher power in panel b*. A photomicrograph of the electrode assembly is shown superimposed on the section and the tip of the tungsten recording electrode is shown by the white arrow. (c) Specific nNOS staining was also observed in small clumps that appear to be axonal swellings and examples are indicated by black arrowheads. (d, e) Show the same coronal section through VCN stained for NADPH‐diaphorase which indicates the presence of all three isoforms of NOS. The large arrowheads indicate darkly stained globular bushy cells while the black arrows indicate darkly stained multipolar cells. The red overlay indicates the presence of blood vessels that surround the NO producing cells. The white arrows indicate faintly stained multipolar cells which are surrounded by a plexus of blood vessels. (f) Example of the excellent signal to noise ratio obtained by the tungsten recording electrodes and the pattern of spikes shown by a primary‐like bushy cell. The red line shows the threshold for spike discrimination and the time of each resolved spike is shown by the dots at the top of the trace. (g–i) Low‐power views of the VCN cut in the coronal plane and stained for Nissl substance, nNOS and thallium uptake. This shows the numbers of neurones at a corresponding point in the posterior VCN stained by the three methods [Colour figure can be viewed at http://wileyonlinelibrary.com]

We examined the antibody staining against nNOS in the VCN. The staining intensity varied greatly between neurones of the same type with some being lightly stained and some being as intensely stained as any found in other parts of the brainstem. Staining was present in the somata, but also extended well into the dendrites. Most were cytoplasmic, but there was also evidence of discrete particles that were associated with the postsynaptic membrane. This particulate staining was found in all cell types and examples are indicated by small black arrows in Figure 2a,b on the dendrites of an OC and on multipolar cells (MC) as well as the soma of a globular bushy cell (GC). Specific staining was also present in small structures that appeared to be axonal swellings (large arrowheads in Figure 2c). Thus, in VCN nNOS may be located either presynaptically, postsynaptically or in the cytoplasm of some cells, but not others. Not all neurones contained nNOS as some were completely unstained. An indication of the proportion of neurones stained is shown by comparing a low magnification view of the VCN stained for nNOS (Figure 2h) with a Nissl stained section (Figure 2g). Some of the neurones are small granule cells that form a granule cell cap over the dorsal aspect of VCN (gr), but these cells do not contain detectable levels of nNOS. We also used the neuronal marker thallium to look at the number and staining variability of neurones in the VCN (Figure 2i). There were more thallium stained neurones than were stained with the nNOS, but like the nNOS, the level of staining was very variable with some neurones having taken up a large amount of thallium and being darkly stained, while others had taken up a small amount of thallium in the loading period and were lightly stained. Comparisons of neuronal numbers stained by the three methods were made by making counts on five corresponding sections from each of two different brains stained with each method (six brains in all). These gave a ratio of approximately 4 Nissl: 2 thallium: 1 nNOS (1885: 1060: 513).

In Figure 2a,b a photograph of a typical electrode assembly is shown at the same magnification as the stained cells to give an impression of how locally the drugs would be released in this study. The white outlined arrow in Figure 2b shows the 10 μm tip of exposed tungsten wire extending beyond the glass insulation. About 30 μm further back from these are the five barrels of the attached micropipettes used for drug administration. With this arrangement, the soma of the recorded neurone should be exposed to relatively high concentrations of drug when they are released iontophoretically. Examples of the spikes recorded by a tungsten electrode of one of these assemblies are shown in Figure 2f for a globular cell that shows a primary‐like with a notch firing pattern. By using the stain NADPH‐diaphorase (reduced nicotinamide adenine dinucleotide phosphate diaphorase) it is possible to visualize all three isoforms of NOS if they are present in a tissue. In addition to the neuronal staining such as the globular bushy cells (large arrowheads in Figure 2d) and MC (Figure 2d black arrows), there are also small dense patches of endothelial NOS associated with the lining of the blood vessels that densely permeate the VCN. These blood vessels are indicated (Figure 2e) by the red overlay that has been superimposed on the same micrograph as shown in Figure 2d. Thus although the NO released from a neurone or exogenous chemical can diffuse readily in the tissue it will rapidly encounter a blood vessel where it will be inactivated by binding to haemoglobin. The dense arrangement of blood vessels appears to form micro‐compartments where there are neurones with low levels of NADPH‐diaphorase (white arrows in Figure 2d) that are isolated from the neurones with relatively high levels of staining. There was no evidence of NADPH‐diaphorase staining in either astrocytes or microglia which is where the inducible form of NOS (iNOS) would be found if there was any pathological damage (Wallace & Bisland, 1994).

### 3.2 Physiological characterization of units

**Figure 3:**
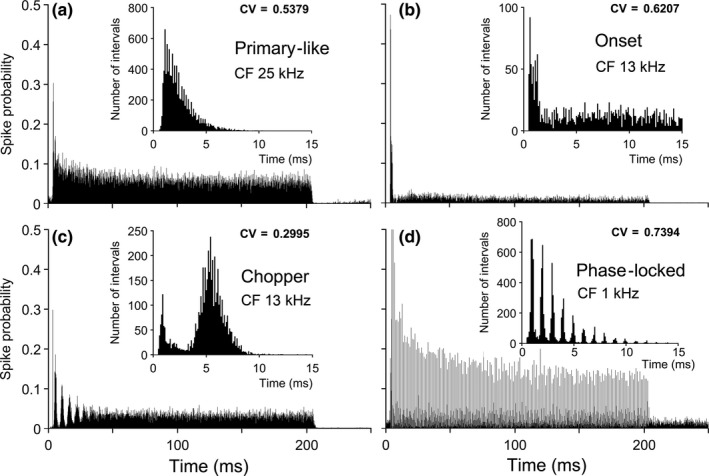
(a–d) Examples of PSTHs from units illustrating the four main subgroups to which units were assigned. The CF of each unit is shown beneath its group name. The inter‐spike interval histogram for each unit is also shown as an insert with the coefficient of variation (CV) in the top right of each panel. The fifth subgroup consisted of the remainder of the units which did not easily fit into one of the above groups

Recordings were made from a total of 27 guinea pigs (17 male, 10 female), resulting in 117 single‐unit recordings that were at least 1 hr long. Units where the spike amplitude fell below the initial threshold level, before the end of the final recovery period, were discarded. After an initial identification of their CF and minimum threshold for activation, all units were stimulated throughout the analysis period with 200 ms tone pips at a frequency corresponding to their CF and at 20 dB above threshold (example in Figure 2f). Using these 200 ms tones we confirmed the unit response type as one of the four main physiological groups in the VCN as illustrated in Figure 3. The units were assigned to the physiological subgroups as follows: 42 Choppers (Ch), 22 Primary‐like (Pr), 15 Phase‐locked (Ph), 18 Onset (On) and 20 Unclassified (Un). The primary‐like units (Figure 3a) showed an initial onset that decayed away rapidly to a steady firing rate and a smooth distribution of inter‐spike intervals (ISIs), while the onset units (Figure 3b) gave one or two spikes at the onset and then the spike probability dropped suddenly to a level close to 0. Their ISIs showed intervals of between 1 and 2 ms between the first two spikes and then a wide but flat distribution of spike intervals thereafter. The chopper units (Figure 3c) had a distinctive, regular pattern of firing at the onset that gradually decayed away to a steady state. The ISI histogram showed a single peak that was usually for values of 2–3 ms followed by a gradually diminishing tail. The phase‐locked units (Figure 3d) showed very regular firing throughout the duration of stimulation and had an ISI that showed regular peaks at multiples of the stimulus waveform period (in this case 1 ms). The response type, as observed in the PSTH pattern, was not altered by administration of any of the drugs used in this study and remained stable throughout the analysis period even although there were sometimes large changes in the firing rate.

No strong associations between unit type and either L‐NAME, SIN‐1, SNOG or NMDA sensitivity were seen (Table 1). Effects observed during drug ejection were not cell‐type specific, as the proportions of unit types responsive to each drug were similar to the proportion of unit types recorded from overall. A Fisher's exact test showed no significant differences between the three main physiological groups (*p* = .284). Each physiological subgroup had at least one example that responded to one of the NO‐related drugs, but the low numbers of units responding to drugs meant that any weak effect would not be found. Overall, around 28% (24/85) of units in the VCN had firing rates modulated by NO‐related drugs (L‐NAME, SNOG or SIN‐1).

**Table 1:**
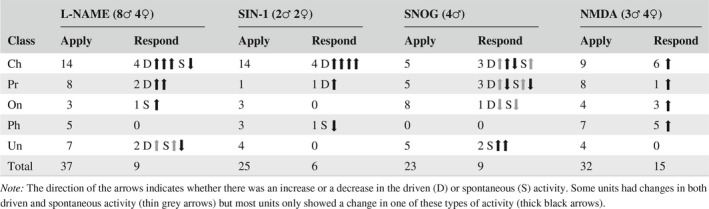
The number of units recorded of each cell type, and the numbers of each unit type found to be responsive to drug applications

### 3.3 Control experiments to confirm the effectiveness of the iontophoretic application of drugs

**Figure 4:**
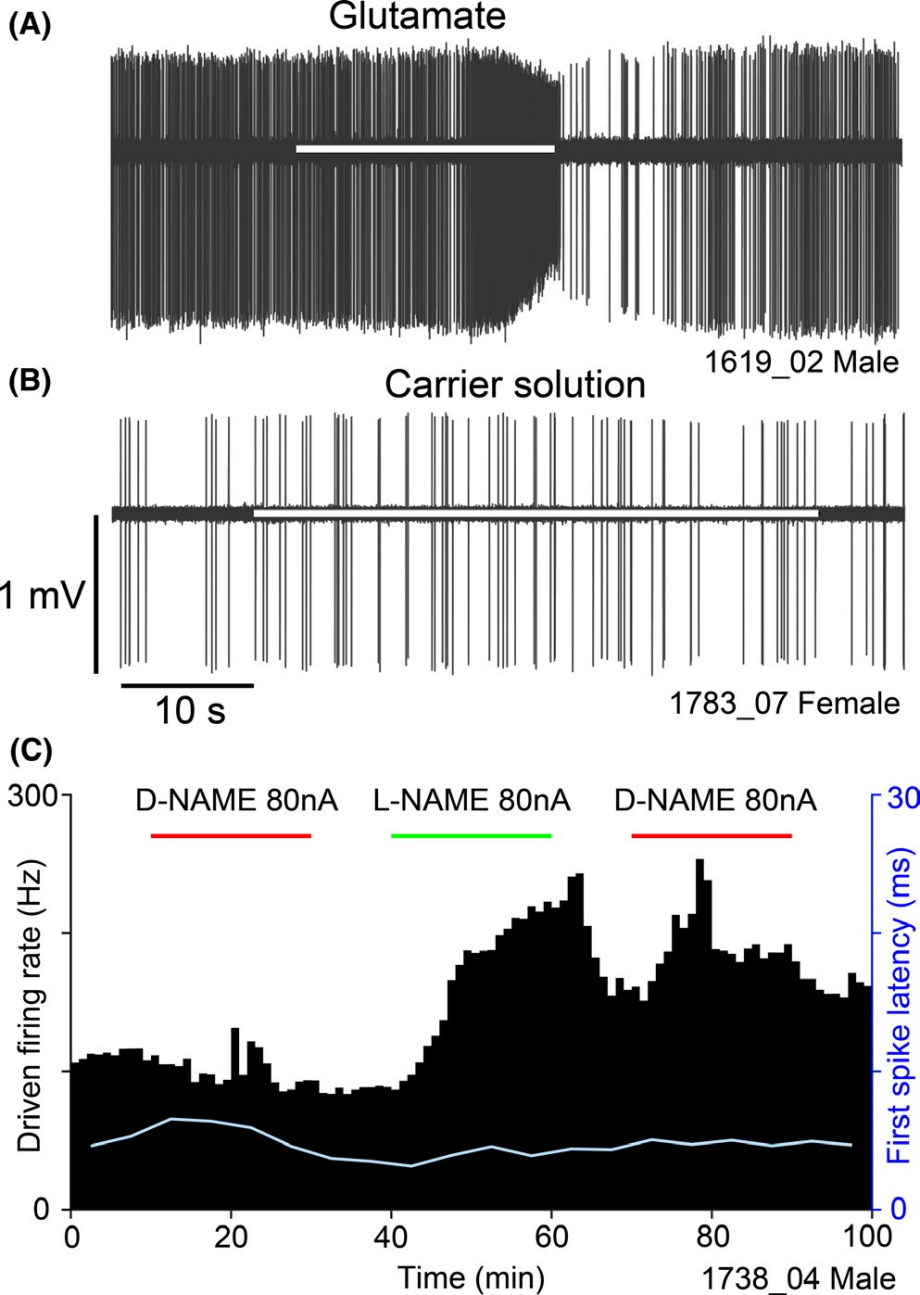
Two single‐unit recordings from VCN that show the spontaneous firing rate and spike amplitude over 1 min. In this and subsequent figures, the unit number is given in the bottom right hand corner of the panel. (a) Glutamate (100 mM, pH 8) was applied for 20 s at 80 nA (as indicated by the white bar) and after about 15 s. the firing rate increased and the spike amplitude started to decrease. (b) A salt solution (100 mM, pH 8) was applied for 40 s at 80 nA (white bar) without any effect on the firing rate or spike amplitude of the recorded unit. (c) A spike‐rate histogram of driven activity of a VCN neurone over 100 min showing no response to D‐NAME (red bars), but an increase in driven firing rate during L‐NAME application (green bar). Bin size 1 min. First spike latency was also recorded (pale line) [Colour figure can be viewed at http://wileyonlinelibrary.com]

At the start of each track within the VCN, checks were made to confirm that the electrode assembly was working effectively before applying the NO‐related drugs. One of the pipette barrels was filled with 100 mM glutamate and current was applied to it (80 nA) to check that the micropipette tips were within effective range of the recording tip of the electrode. Most units responded to glutamate with an increase in firing rate within 20 s of the current being switched on. An example of this is shown in Figure 4a where glutamate was applied to a unit with a moderate spontaneous firing rate (8 sp/s) for a total of 20 s. The firing rate increased within 15 s of the current being applied and remained high while the current was on. When the firing rate increased the spike amplitude progressively decreased. It then recovered to its previous amplitude and spontaneous firing rate over the next 20 s. The effects of glutamate were not quantified as its effects are well known and were not part of this study. However, they provided a useful comparison for control experiments where buffered saline with the same pH as the NO‐related drugs was applied from an adjacent pipette barrel at the same current strength (80 nA). An example of this is shown in Figure 4b where a salt solution (100 mM, pH 8) was applied to a unit with a low spontaneous firing rate (1 sp/s) for 40 s without any effect on either firing rate or spike amplitude. We never saw any significant effect of current application using a salt solution with pH values of 4.5 or 8 using citrate or phosphate buffer and currents of 80 nA.

**Figure 5:**
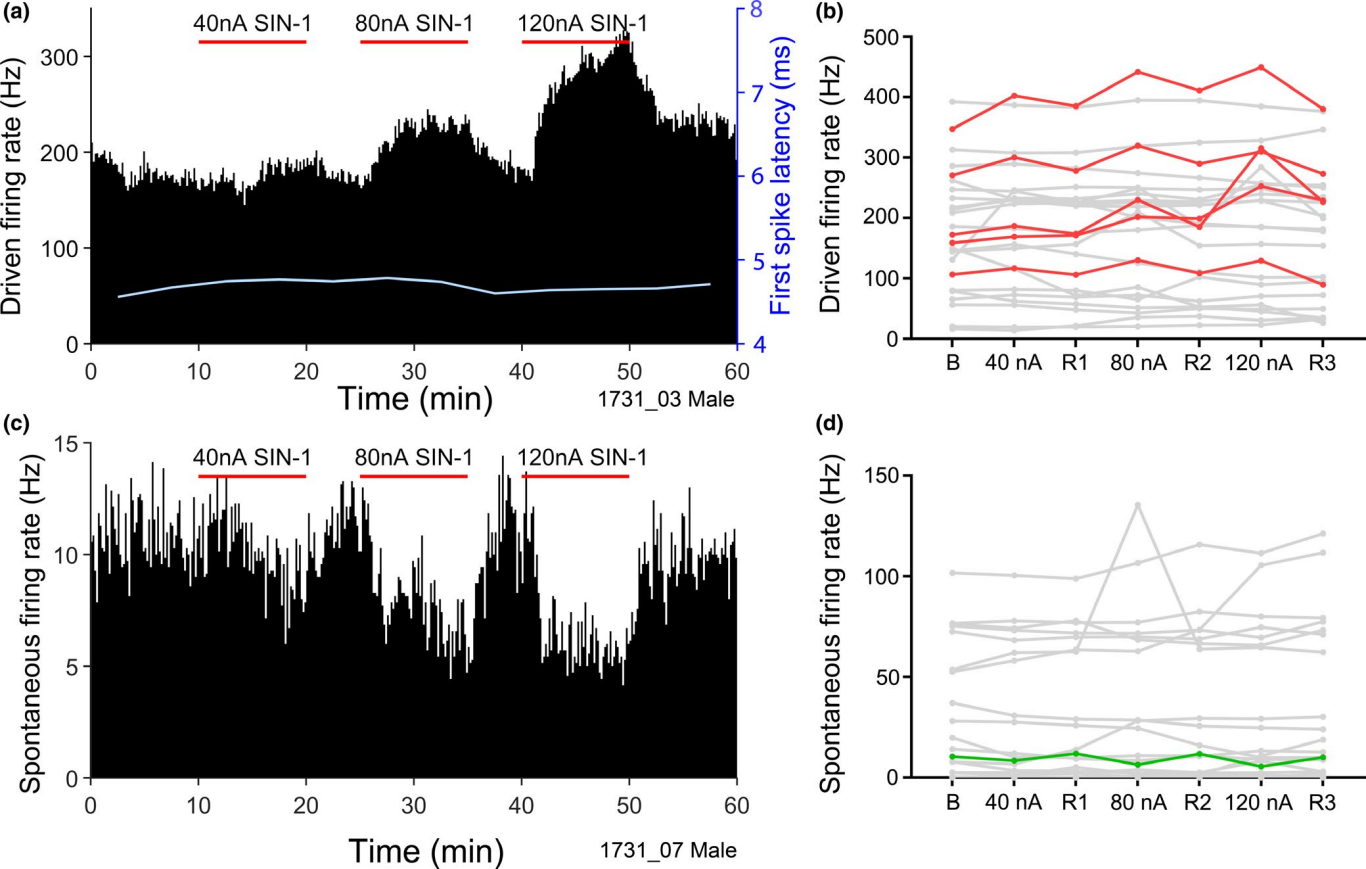
(a, c) Spike‐rate histograms (bin size 10 s) of two VCN units over 1 hr. Three periods of SIN‐1 ejection at varying strength are indicated by the red horizontal bars. (a) Driven firing rate for a chopper unit with CF of 3 kHz. For driven firing rate, the first spike latency (FSL) averaged every 5 min is shown by the pale line. (c) Spontaneous firing rate for an Unclassified unit with CF of 12 kHz. (b, d) Mean driven and spontaneous firing rates of 25 VCN units during SIN‐1 application. Each line is data for one unit. The red lines in (b) and the green line in (d) show units in which the changes in spike rate with SIN‐1 application were statistically significant [Colour figure can be viewed at http://wileyonlinelibrary.com]

The biologically inactive isomer of L‐NAME, D‐NAME, was also used as a control. Over 100 min, 50 mM D‐NAME and L‐NAME were each applied at 80 nA for 20 min. This protocol was carried out on 15 VCN neurones from three animals (all female). L‐NAME caused a significant and reversible increase in driven firing rate in only two of these units and an example is shown in Figure 4c. By contrast, D‐NAME failed to produce a significant response in these two or any of the other units tested. In the example shown in Figure 4c there do appear to be small transient increases in firing rate during both the D‐NAME application periods. However, these appear to just be random fluctuations in the firing rate that were also present when no drug was being applied. Random fluctuations of this sort were also present in other recorded units such as a few of those shown in Figure 5b. The periods analysed were the last 10 min of the response to D‐NAME and the firing rates then were not significantly different from the first and last 10 min of the recording in Figure 4c. Thus only the L‐NAME application produced any significant changes.

### 3.4 Firing rate changes in response to application of NO Donor (SIN‐1)

Figure 5 demonstrates that application of the NO donor SIN‐1 caused dose‐dependent increases and decreases in firing rate of VCN units. Figure 5a shows the firing rate of a unit, in response to the CF tone in 10‐s bins over 60 min, during which SIN‐1 was applied with three different ejection currents. It is clear that the SIN‐1 evokes an increase in the driven discharge rate at all three ejection currents. It is also clear that the more SIN‐1 that is ejected the greater the increase in discharge rate with no indication that this effect has saturated as the 120 nA ejection current produced a much larger increase than did the 80 nA. The firing rate increase develops slowly: it increases over the first 5 min or more of drug ejection and then stabilizes at a level that is ejection current dependent. After cessation of the ejection current the firing rate increase also subsides over several minutes. Figure 5c shows that, in other units, SIN‐1 can cause decreases to the spontaneous firing rate, that mirror those described above and show a similar ejection current dependency. The changes build up and subside over similar time courses (i.e. over several minutes).

SIN‐1 was applied to a total of 25 units from four animals (two male, two female). The effect of SIN‐1 on the driven firing rate of all these units is shown in Figure 5b. In this figure, each grey line indicates the data for one unit and the red lines show the data for the 5/25 (20%) units that showed significant increases in discharge rate. There were no units with a significant decrease in driven rate. The fact that the driven firing rate does not recover fully to the starting baseline between current injections again shows that the effects are of long duration. Figure 5d shows changes in the spontaneous firing rate as a result of SIN‐1 application. Again, as for the driven rates, SIN‐1 evoked both increases and decreases in spontaneous rates in different units, but the changes were only consistent and hence significant for one unit where there was a reduced firing rate during drug application for all three drug concentrations (shown by the green line).

During the 120 nA ejection, the mean driven firing rate of the five units that increased went from a mean baseline rate of 211 to 291 Hz (increased to 141.6% of baseline; *SD* = 28.9). These units were of two different types (4 Ch with CFs of 2.5, 3, 8 and 12 kHz, 1 Pr with CF of 9 kHz; Figure 5b: red lines). A decrease from a baseline of 10.5 to 5.5 Hz (52.3% of baseline spontaneous firing rate) during 120 nA ejection of SIN‐1 was observed in one unit (Ph with CF of 1.6 kHz; Figure 5d green line). The first spike latency (FSL) was never affected by SIN‐1 application. An example is shown by the pale line in Figure 5a.

**Figure 6:**
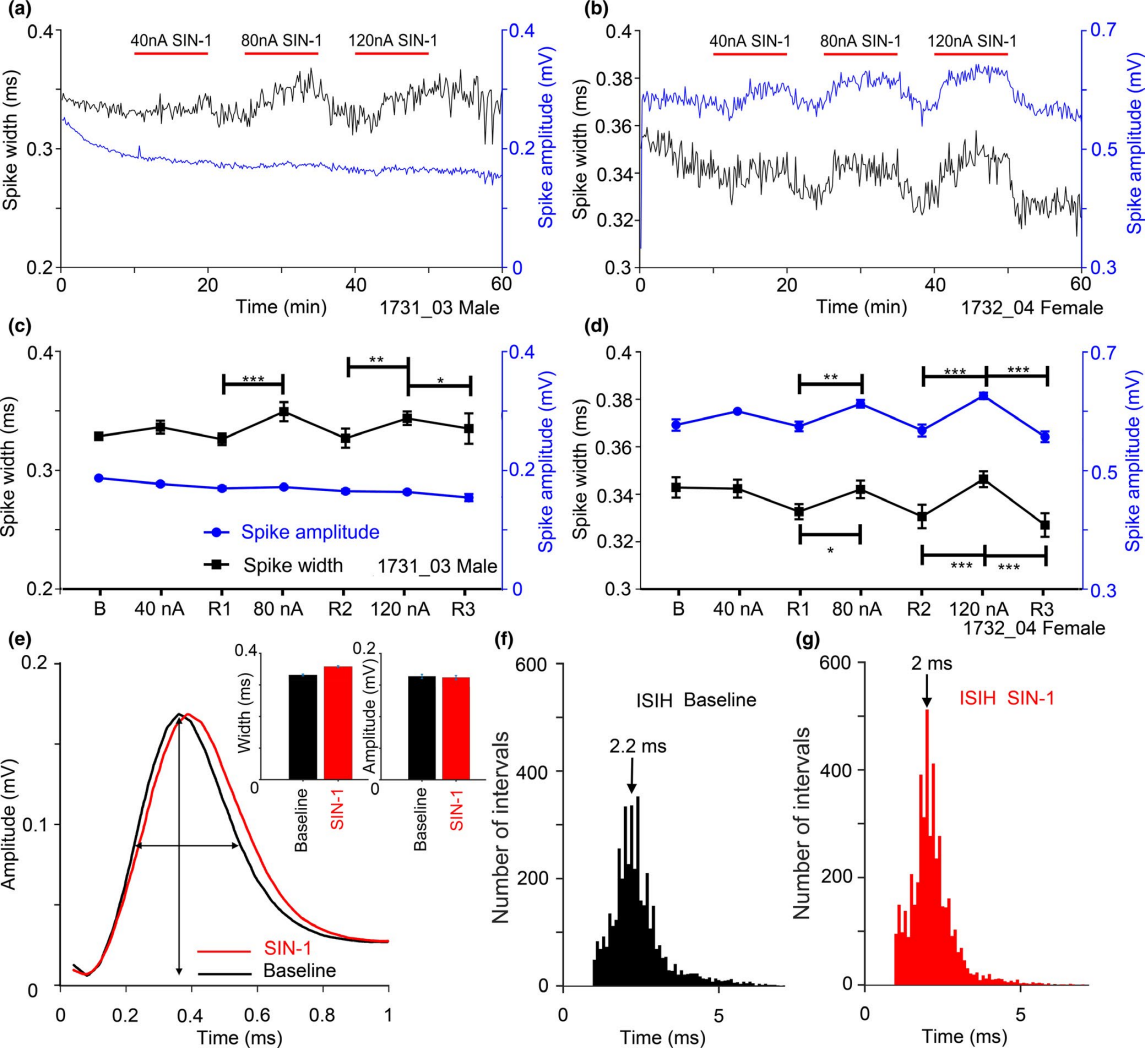
(a, b) Spike amplitude (blue lines) and spike width (black lines) of two VCN units recorded over 1 hr, during three periods of increasing strength of SIN‐1 application. (c, d) Quantification of the increases observed in (a, b), respectively, with the data shown as the mean ± the standard error based on 10 s analysis periods as described previously. Comparisons between analysis periods were made using a Kruskal–Wallis test with Dunn's multiple comparison test. Significant changes are indicated by the presence of one or more asterisks. (d) Shows a significant increase in spike amplitude and width during SIN‐1 application, while (c) displays increased spike width with no alteration to spike amplitude. (e) Mean waveform of spikes during 5 min. of the baseline period and the last 5 min. of the SIN‐1 application at 80 nA for the unit shown in (a). The double‐headed arrows indicate the amplitude and width of the baseline spike. (f, g) Inter‐spike interval histograms of the unit shown in (b) during the second recovery period and the SIN‐1 application at 120 nA. The arrows mark the median values based on the first 20 ms of each response [Colour figure can be viewed at http://wileyonlinelibrary.com]

To look for evidence that NO affects postsynaptic events, the shape of the extracellular spike was analysed for all data. The extracellular spike is an indirect measure of transmembrane potential, but should still reflect changes in membrane currents. The NO donor SIN‐1 produced an increase in the width of the extracellularly recorded spike shape in two chopper units (Figure 6) and both units showed increased driven firing rates in response to the SIN‐1. The first unit (Figure 6a) showed an increase in width (black line) in the presence of SIN‐1, while the amplitude was unaffected (blue line). However, the second unit (Figure 6b) showed a correlated increase in both width and amplitude when SIN‐1 was applied. The effect of SIN‐1 on the first unit seemed to be maximal at an application level of 80 nA as increasing the current to 120 nA did not produce a bigger effect (Figure 6c). By contrast, in the second unit there was a monotonic increase in both the width and height of the spike as the SIN‐1 levels increased (Figure 6d). The change in the shape of the spike illustrated in Figure 6c is shown in Figure 6e which is the mean shape averaged over a period of 5 min. The slope of the depolarizing phase is less steep during the SIN‐1 application, implying that the released NO affected the currents at the start of the spike (possibly Na_V_ or K_V_1). In addition, the repolarizing phase of the spike is also delayed and this is consistent with an effect on voltage‐dependent potassium channels. Both of these units showed chopper responses, and we studied the ISI distribution of the first 20 ms of the response during the recovery period compared with the response during the end of the SIN‐1 application. For both units the median ISI decreased during the SIN‐1 application: for the second unit, the value decreased from 2.2 to 2 ms (Figure 6f,g). This change was significant for the second unit as shown by a two‐sample Kolmogorov–Smirnov test (*p* = .0436).

### 3.5 Firing rate changes in response to application of NO donor (SNOG)

**Figure 7:**
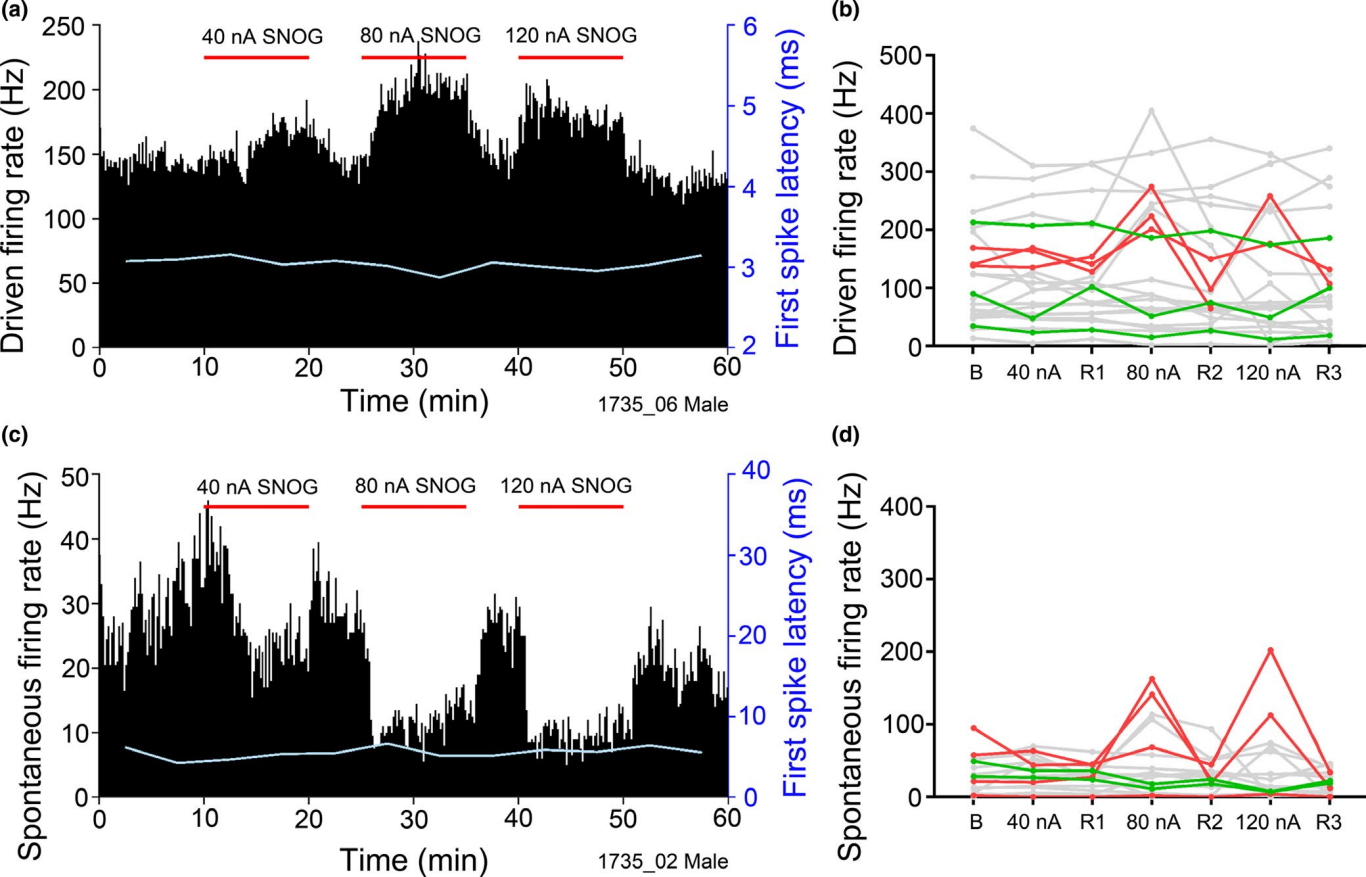
(a, c) Spike‐rate histograms (bin size 10 s) of two VCN units over 1 hr. Three periods of SNOG ejection at increasing strength are indicated by the horizontal bars. (a) Driven firing rate for a chopper unit with CF of 17 kHz. The first spike latency averaged every 5 min is shown by the pale line. (c) Spontaneous firing rate for a primary‐like unit with CF of 12 kHz. (b, d) Mean driven and spontaneous firing rates of 23 VCN units during SNOG application. Each line is data for one unit. The darker lines show units in which the changes in spike rate with SNOG application were statistically significant [Colour figure can be viewed at http://wileyonlinelibrary.com]

A total of 23 single units were recorded from during the iontophoretic ejection of SNOG and the results are summarized in Table 1. Nine units (39%) showed a significant change in driven and/or spontaneous firing rate and all the main morphological types of neurone were affected. Both significant increases and decreases in the driven firing rate of chopper and primary‐like neurones were observed along with a decrease in the driven rate of an onset neurone and increases in two neurones of unspecified type. Of the six neurones showing a significant change in driven rate, three showed a mean increase from a baseline of 149 Hz to a rate of 256 Hz with the 120 nA injection level (170% increase, *SD* 38.8) and three showed a mean decrease from a baseline of 112 Hz to a rate of 78 Hz with the 120 nA injection level (56.3%, *SD* 24.7). An example of SNOG increasing the driven rate of a chopper neurone is shown in Figure 7a. There was a significant effect of the SNOG at all three concentrations, but in this and other neurones (Figure 7b), the effects seemed to saturate at about 80 nA. Increasing the current to 120 nA did not significantly increase the driven rate compared with 80 nA in any of the neurones. Spontaneous rate was also significantly altered by SNOG in the three main morphological types with both increases and decreases being observed in six neurones (Figure 7d). Of this six, four showed a mean increase from a baseline rate of 44 Hz to a rate of 163 Hz at an injection level of 120 nA (378%, *SD* 202.4) while two showed a decrease from a baseline of 39 Hz to a rate of 7 Hz at a level of 120 nA (20.2%, *SD* 7.4). Three of the units showed changes in both driven and spontaneous firing rates. An example of a primary‐like neurone where the spontaneous rate is significantly decreased by SNOG application is shown in Figure 7c. We did not record from any neurones with phase‐locked responses at CF and so did not check for an effect of SNOG on temporal precision but we did check for first spike latency changes. The first spike latency did vary by about ±10% over the recording period but there was never any significant correlation between first spike latency and SNOG application in any of the neurones.

### 3.6 Firing rate changes as a result of application of NOS inhibitor L‐NAME

**Figure 8:**
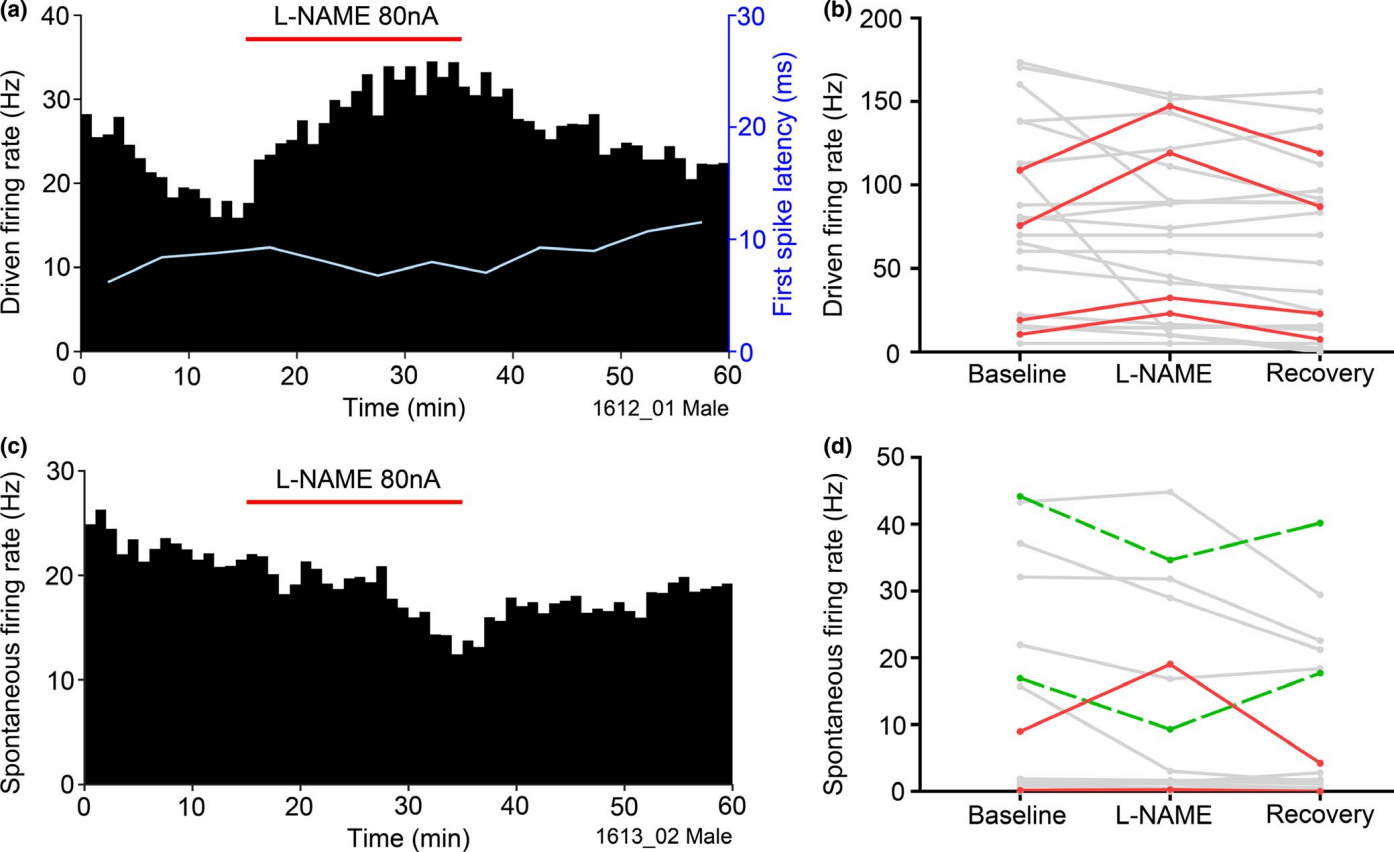
(a, c) The driven and spontaneous spike‐rate histograms of two VCN single units, showing an increased driven rate (a; Ch with CF of 12 kHz) and decreased spontaneous rate (c; Un with CF of 10 kHz) in response to iontophoretic L‐NAME application. Bin size 1 min, 80 nA L‐NAME application is indicated by the red bar. The first spike latency (FSL) of the driven rate is shown by the blue line. (b, d) Mean driven and spontaneous firing rates of all 22 VCN units. Each grey line is the data for a single unit. Units showing statistically significant increases in spike rates are shown as darker continuous lines while those showing statistically significant decreases in spontaneous rate are shown by the dashed dark lines [Colour figure can be viewed at http://wileyonlinelibrary.com]

The exogenous NO provided by the SIN‐1 or SNOG supplements the NO that is already being produced by the endogenous nNOS, and so, the neurones will potentially be exposed to unnaturally high levels of NO. An alternative way of looking at the physiological effects of NO on the recorded neurones is to block the endogenous nNOS. Thus measurements of firing rate over a period of 1 hr were used to identify the effect of the NOS inhibitor L‐NAME on VCN neurones. A total of 22 complete recordings, using L‐NAME alone, were made in nine animals (eight male, one female). L‐NAME clearly produced a change in both driven and spontaneous firing rates in a subpopulation of neurones, as shown in Figure 8. Over the course of the 1‐hr recording, only one application of L‐NAME was given and the firing rates were measured over 1‐min intervals. It is clear from the example shown in Figure 8 that the effect of L‐NAME on the driven rate takes a long time (20 min) to develop fully and a similar amount of time to dissipate. Out of the 22 units tested 4 (18%) showed a significant increase in firing rate during L‐NAME application and this effect was reversed when the drug was no longer applied (dark lines in Figure 8b). The rise in the driven rate during L‐NAME application was quite large, with firing rates rising from a mean baseline of 53.6 Hz to a mean of 80.5 Hz (170.4%; *SD* = 35.3). The cell types and characteristic frequencies of these 4 neurones were: Ch, 6 kHz; Ch, 12 kHz; Pr, 2.6 kHz; Un, 10 kHz. Two units also showed a large decrease in the driven rate after the L‐NAME was applied, but this effect did not reverse when the drug application stopped and thus we could not be sure that the reduction of the driven rate was a specific effect of the drug. There were also effects on the spontaneous rate and an example of a significant reduction is shown in Figure 8c where the change follows a similarly extended time course to the change in the driven rate described above. Out of the 22 single units recorded 2 (9%) showed a significant increase in mean baseline spontaneous rate of 4.6 to 9.7 Hz (to 178.9% of baseline, *SD* 47.7) during L‐NAME ejection and 2 (9%) showed a significant decrease in spontaneous rate from a baseline rate of 30.6 Hz to a rate of 22 Hz (66.6% of baseline, *SD* 16.7). In all of these cases, the spontaneous rate returned to a level close to the baseline after the drug application was stopped (Figure 8d). The cell types and characteristic frequencies of these responsive neurones were: spontaneous rate increases (On, 12 kHz; Un, 10 kHz) decreases (Ch, 14.25 kHz; Un, 9.5 kHz). Changes in the driven firing rates on application of L‐NAME correlated with FSL: as driven firing rate increased, FSL decreased (Figure 8a, blue line). One of the unclassified units (Un) showed an increase in both driven and spontaneous activity (Table 1) but all the other affected units only showed a change in either the driven or spontaneous activity. Table 1 shows the combined units from the group where only L‐NAME was applied and the group where both L‐ and D‐NAME were applied.

**Figure 9:**
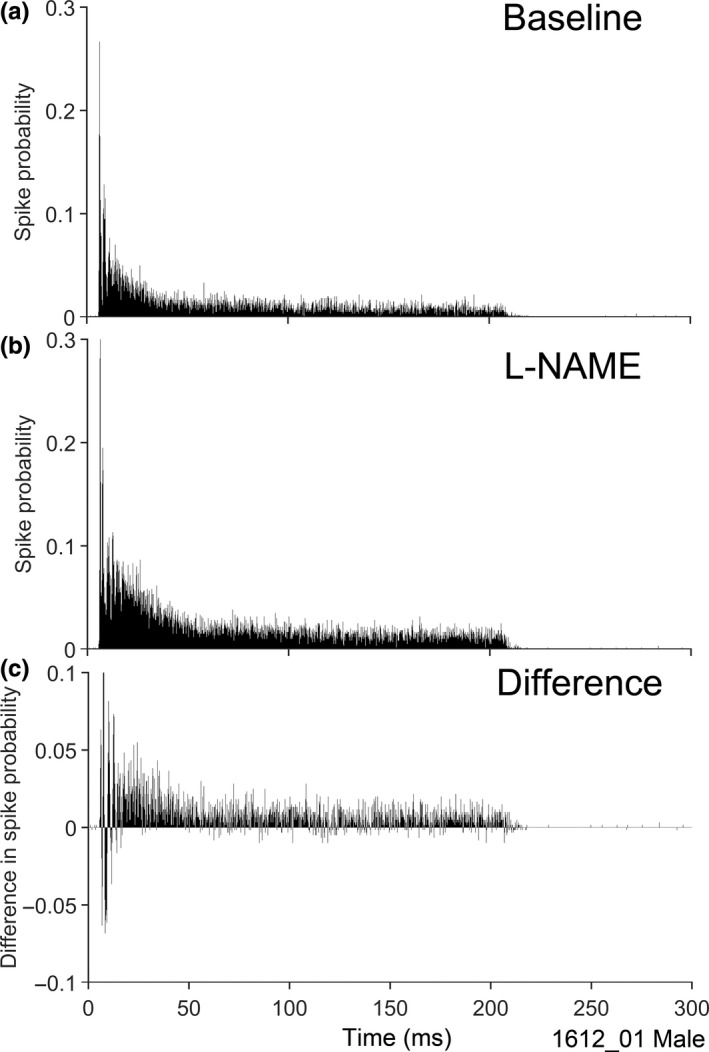
(a) PSTH of the chopper unit from Figure 4a over the baseline period (10 min, sweeps 301–900; bin size 0.2 ms). (b) The PSTH of the same unit during L‐NAME application (10 min, sweeps 1,501–2,100; bin size 0.2 ms). (c) The change in this PSTH during L‐NAME application (top–middle). A two‐sample Kolmogorov–Smirnov test used to compare between the distributions showed they were significantly different (*p* < .01)

To determine whether the L‐NAME had its effect throughout the duration of the CF‐tone‐driven response, the PSTHs during L‐NAME application were subtracted from the baseline PSTHs to calculate the difference in spike probability. An example set of baseline (Figure 9a) and L‐NAME (Figure 9b) PSTHs and the difference PSTH (Figure 9c) for a typically responsive unit shows that the facilitatory effects of the localized reduction in NO are slightly stronger during the first 50 ms of the response, but they continue throughout the duration of the tone pip. Large fluctuations in difference in spike probability are seen in the initial 20 ms of the driven period, likely due to a slight decrease in first spike latency altering the timing of the chopping pattern. The population data showed that, for all units in which the driven rate was affected by L‐NAME, the facilitation continued throughout the 200 ms driven period.

### 3.7 Neither the NO donor SIN‐1 nor the NOS inhibitor affects phase locking

**Figure 10:**
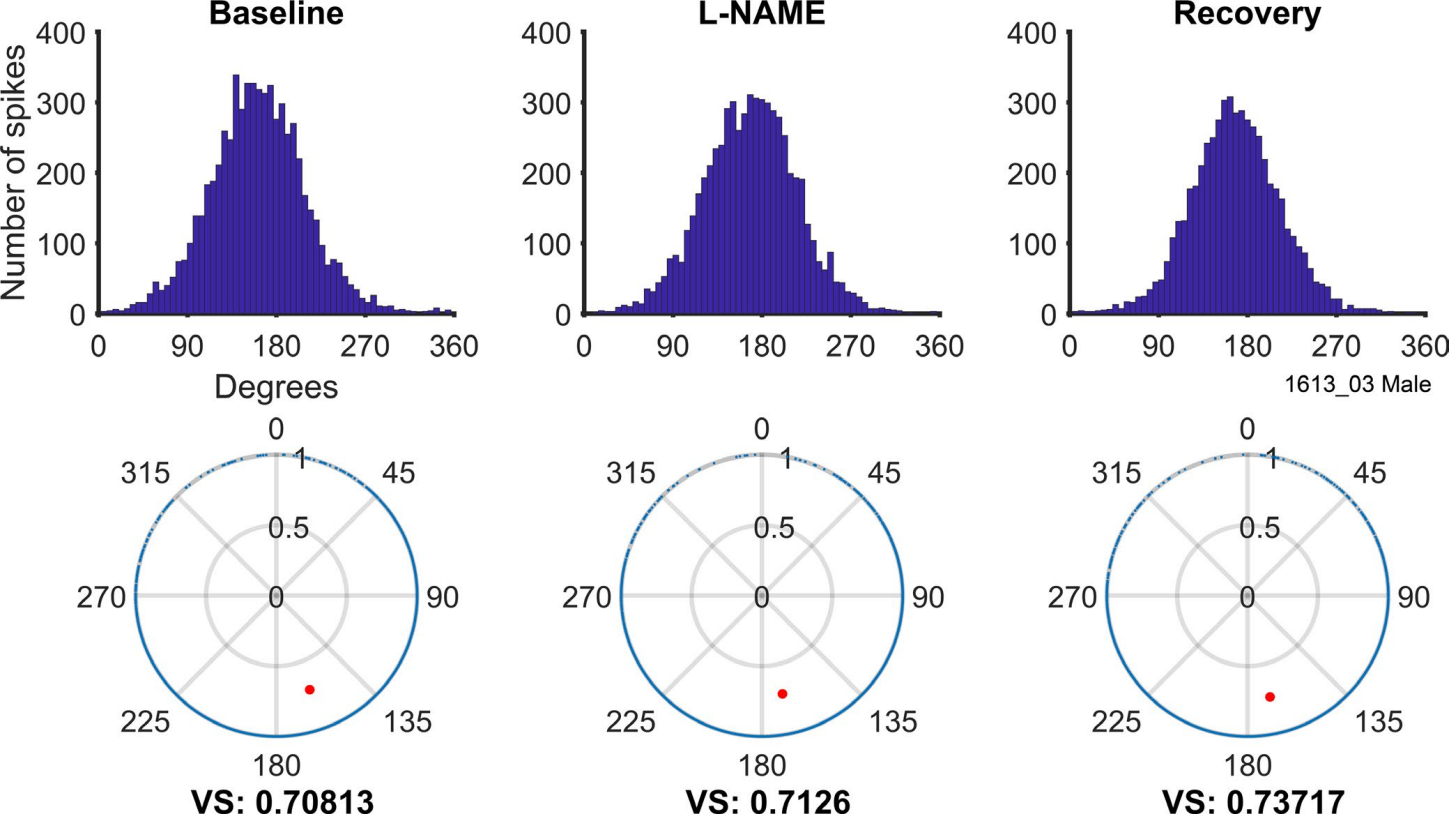
Phase‐locking analysis of a 400 Hz unit during the three analysis periods: Baseline, L‐NAME and Recovery (columns left to right). Top row: Period histograms for spikes during each of the three analysis periods. Bottom row: polar plots showing individual spikes as blue dots around the edge. Vector strength (VS; length along radius) and characteristic phase (degrees) are indicated by the red circle. No changes in vector strength or timing are observed between the three analysis periods [Colour figure can be viewed at http://wileyonlinelibrary.com]

Low‐frequency units in the VCN are able to provide precise timing information about the period of a sound stimulus by locking their spikes to a particular phase of the sound wave. Changes in membrane conductances that might be produced by NO receptors could interfere with both the phase and strength of this temporal coding and thus we sought evidence for these types of changes. Phase locking was only identified by stimulating units at CF (not in their low‐frequency tail) and was tested for all units with a CF below 5 kHz. A total of six units with phase‐locked responses were tested in the presence of L‐NAME and a further six in the presence of SIN‐1. The first row of Figure 10 shows the distribution of spikes in a period histogram for a unit with excellent phase locking; a vector strength of over 0.7. This phase locking was unchanged by the application of L‐NAME. The lower row of Figure 10 shows the vector diagrams with the vector strength and phase angle indicated by the single dot. It is clear from these plots that L‐NAME did not change the magnitude nor the phase of the response of this unit. None of the 12 units showed significantly altered phase locking due to either drug application.

### 3.8 Inhibition of NOS during NMDA‐evoked excitation

**Figure 11:**
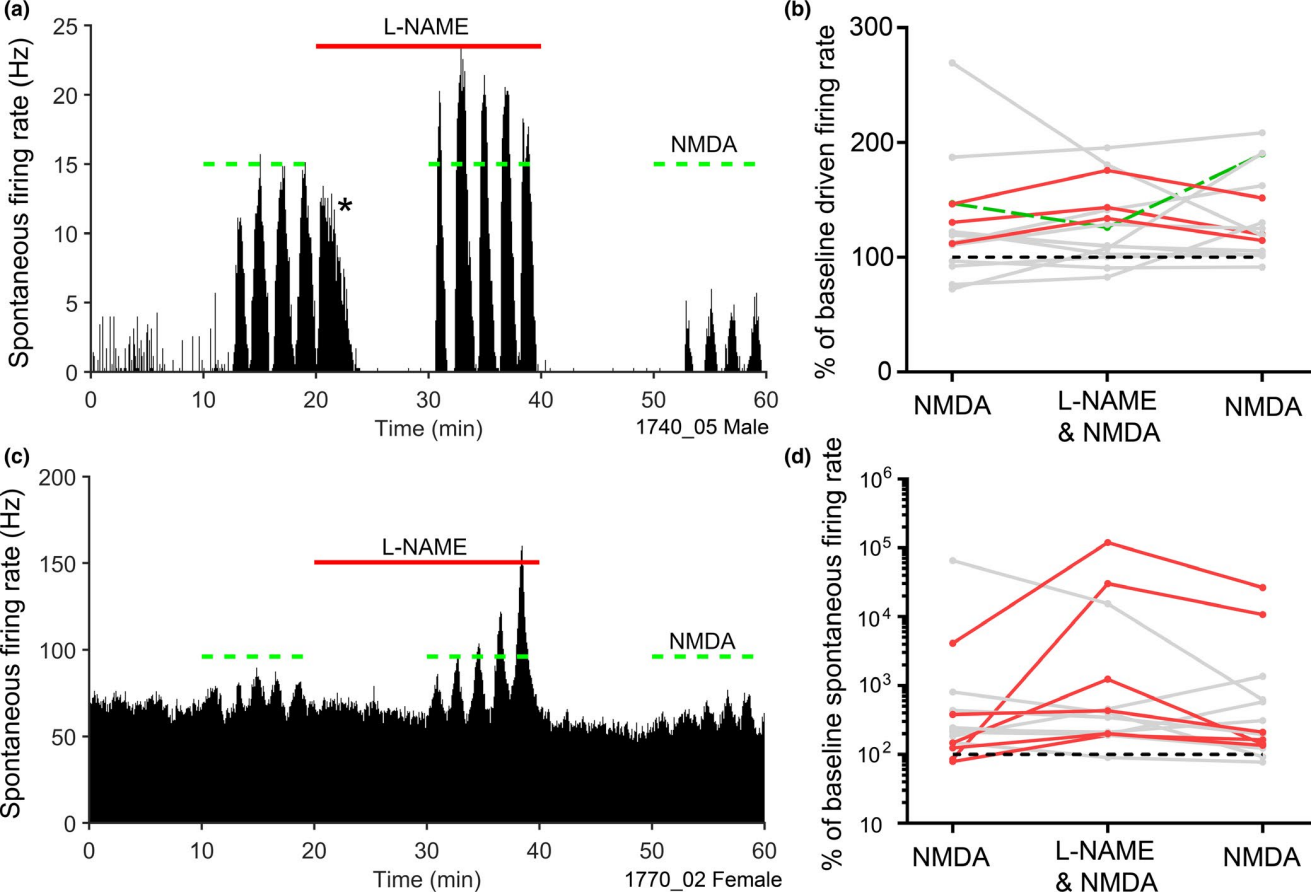
(a, c) Spike‐rate histograms of two VCN units which both displayed an increase in spontaneous firing rate during 1 min bursts of NMDA application (green bars). Enhancement of this NMDA‐evoked excitation occurred during the 20‐min L‐NAME application (red bars) for units with low (a) and high (c) spontaneous rates. Bin size = 10 s. (b, d) The percentage of baseline driven and spontaneous firing rates evoked by NMDA bursts before, during and after L‐NAME application. Log% was used for spontaneous firing rate increases due to the very low baseline firing rates in some units. Significant increases and decreases are highlighted by red and green lines, respectively. Black dotted line marks 100% (no change) [Colour figure can be viewed at http://wileyonlinelibrary.com]

Glutamate is the main excitatory transmitter in the VCN but there are different types of ionotropic and metabotropic receptors for glutamate in addition to the NMDA receptors that can be directly linked to nNOS (Collingridge & Lester, 1989). In order to confirm that NMDA receptors are important in mediating the effects of NO in the VCN, NMDA was applied directly by iontophoresis along with the NOS blocker L‐NAME. The effect of L‐NAME on NMDA‐mediated excitation was characterized in 32 recordings from seven animals (three male, four female; Table 1). NMDA evoked an increase in the spontaneous firing rate in 15/32 (47%) of units with no correlation with cell type or initial spontaneous rate. The NMDA was introduced in trains of five applications, each lasting 1 min. The first application of current usually had little or no effect, and even in responsive units, there were often just four bursts of increased activity as shown in the last 10 min of Figure 11a. These bursts of increased spontaneous activity were enhanced by the application of L‐NAME in six units (40%; darker lines Figure 11d). These increased firing rates were seen in both low (Figure 11a) and high spontaneous rate units (Figure 11c). In three units, the application of L‐NAME immediately following the initial NMDA bursts induced firing rate increases at the onset of the L‐NAME even when the NMDA was no longer being applied (seen in Figure 11a as activity marked by the *).

Of the 15 units that were NMDA‐sensitive, L‐NAME application enhanced NMDA excitation during the driven period in 3 (20%) units and reduced excitation in 1 (7%; Figure 11b). The effects of L‐NAME on some individual neurones were quite large but overall L‐NAME only had an effect on a small proportion of neurones (9/37; 24%).

## 4 DISCUSSION

The effects of NO on the activity of single neurones in the VCN has not been studied in vivo before, although there has been a recent study on patch clamped neurones in mouse brain slices (Cao et al., 2019). Thus our main purpose was to initially establish what effect NO has in the intact VCN rather than to investigate the mechanisms of action. The main result was that only about 25% of neurones in the VCN responded to a change in NO levels. This roughly corresponds to the proportion of neurones that contain measurable amounts of nNOS. However, the link between these two numbers is not immediately apparent. At physiological levels, the NO is thought to act on its main receptor which is guanylyl cyclase (Garthwaite, 2019). The distribution of nNOS and guanylyl cyclase may not be the same. Their distribution in the VCN has been compared in the rat and most principal neurones contained both nNOS and guanylyl cyclase, except for OC which apparently did not contain guanylyl cyclase (Burette et al., 2001). The auditory nerve fibres also lacked significant amounts of guanylyl cyclase and this implies that NO would not be acting as a retrograde messenger via cGMP. Thus although spiral ganglion neurones are known to contain nNOS (Burette et al., 2001; Fessenden et al., 1999) and we saw evidence of nNOS in presynaptic terminals, NO produced by the nerve fibres may have a different target. One such target is hyperpolarization‐activated, cyclic nucleotide‐dependent membrane channels (HCN1 Kopp‐Scheinpflug et al., 2015) which can be modified independently of the cGMP pathway.

In the VCN, nNOS is mostly located postsynaptically and, although it mainly appeared to be cytoplasmic, there was also evidence of small patches on the postsynaptic membrane. There are three alternative splice forms of nNOS (α, β, γ) that are characterized by different exons (Lee, Cai, Hubner, Lee, & Lindpaintner, 1997). The major type in the VCN is the β form (Eliasson et al., 1997), which is located in the cytoplasm and lacks an initial pdz domain that would allow it to bind to the postsynaptic density protein (PSD‐95 Brenman, Xia, Chao, Black, & Bredt, 1997). However, there is also some of the α variant which does have an initial pdz domain and can bind to the PSD‐95 protein along with the NMDA receptor to form a multi‐protein complex that includes guanylyl cyclase (Olthof‐Bakker et al., 2019). In our study, almost half of the neurones responded to exogenous NMDA and seemed to have functional receptors. The membrane‐bound nNOS can be activated by calcium entering via the NMDA channel to give a localized increase in cGMP. One of the nearby membrane targets for cGMP is a voltage‐dependent potassium channel (K_v_3.1) which is present in bushy cells (Rusznák et al., 2008) and some stellate cells of the VCN (Perney & Kaczmarek, 1997). NO is a modulator of potassium channels (Artinian,Tornieri, Zhong, Baro, & Rehder, 2010) and they regulate the excitability of VCN neurones (Cao, Shatadal, & Oertel, 2007; Rothman & Manis, 2003).

NO was unequivocally shown to have an action in the auditory nervous system in two papers by Steinert et al. (2008, 2011). In the first paper, they demonstrate that NO inhibition of Kv3 potassium channels in the MNTB produces a widening of the action potential (Steinert et al., 2008), which in turn results in more sustained depolarization, and inactivation of voltage‐gated sodium channels, hence reducing gain and information transmission. In the subsequent paper (Steinert et al., 2011), the authors show that as well as suppression of the Kv3 channels, Kv2 channels are facilitated. This raises spiking threshold and increases fidelity (and hence rate) of firing by reducing failures. Kv2 channels seem, at the very least, to be much less extensively expressed in VCN, but a very wide range of other potassium channels are certainly present (Friedland, Eernisse, & Popper, 2007). Despite this, we only found evidence of either spike widening or change in spike amplitude in two out of 94 neurones where NO levels were manipulated. Both of these neurones showed chopping responses and there may be a significant association between the distinct membrane channels associated with chopping (Oertel, Wright, Cao, Ferragamo, & Bal, 2011) and the NO induced change in spike shape. One of the neurones showed a decreased slope during the depolarization phase of the spike and this is consistent with an effect of NO on sodium channels as shown in the MNTB (Steinert et al., 2008). Despite the widening of the spike shape both of these neurones also showed a decrease in the median ISI during the initial chopping phase that is consistent with an increased firing rate in response to acoustic stimuli. By contrast, none of the neurones with phase‐locked responses showed any NO induced change in temporal fidelity or spike shape.

Most of the nNOS in the VCN is located in the cytoplasm of the principal cell somata (Coomber et al., 2015). The cytoplasmic nNOS may also be activated by calcium from the NMDA channels or released from other sources following depolarization (Garthwaite, 2008; Steinert et al., 2011). The most striking effect of endogenous NO on VCN neurones was the suppression of spike rate during acoustic driving where 16% (6/37) of neurones showed a mean increase of 170% in driven firing rate when NO synthesis was inhibited by applying L‐NAME. This appears to represent a feedback inhibition of NMDA receptors which has previously been shown to be long‐lasting (Manzoni et al., 1992) and may involve S‐nitrosylation (Ahern, Klyachko, & Jackson, 2002; Chen, Jin, & Pan, 2017). The levels of NO produced by nNOS are not normally enough to produce nitrosylation (Garthwaite, 2008), but the dense cytoplasmic nNOS and high mean rates of firing apparently can lead to a build‐up of NO levels sufficient to directly modify protein thiol groups. Even in silence some auditory nerve fibres have spontaneous rates of up to 120 Hz (Tsuji & Liberman, 1997) and with NO having a half‐life of between 7 and 630 ms (Garthwaite, 2019; Santos, Lourenço, Ledo, Barbosa, & Laranjinha, 2012) there could still be a steady build‐up of NO levels as shown in slices of the SOC (Steinert et al., 2008). This explanation is strengthened by the results from the direct application of NMDA where some neurones showed a higher firing rate in response to the NMDA when L_NAME was inhibiting the endogenous production of NO compared with baseline levels. This presumably reflects an effect of endogenous NO similar to the reduction in spontaneous rate induced by SIN‐1 (Figure 5c), that is endogenous NO was holding the spontaneous firing rate down and the inhibition of its production by L‐NAME enables a larger response to the NMDA. In Figure 11a the NMDA responses are reduced following the cessation of L‐NAME possibly reflecting a recovery of the effectiveness of the endogenous NO.

Different mechanisms of action must be involved when exogenous NO (SIN‐1 or SNOG) were applied as they were capable of increasing or decreasing both driven and spontaneous activity. Unfortunately, given the long time course of the effects of the applied drugs, we were unable to apply SIN‐1 or SNOG and L‐NAME sequentially to the same neurone. Thus, we do not know if the neurones which increased their firing rate in the presence of L‐NAME (reduced NO levels), would also have increased their firing rate in the presence of a NO donor. It is possible, and indeed likely, that the neurones with opposite responses to NO formed separate populations. We divided our cells into a minimum number of classes but bushy cells (Cao et al.,^2007^), onset cells (Arnott et al., 2004) and choppers (Palmer et al., 2003; Rhode et al., 1983) can all be subdivided into different subclasses in terms of both their physiological properties and axonal projections. They can also differ in neurotransmitter type: one type of small stellate cell with chopper responses is thought to be glycinergic (Ngodup & Trussell, 2019) while most choppers are thought to be glutamatergic. The T‐stellate chopper cells of the mouse VCN form a bidirectional, positive feedback network that is dependent on NO (Cao et al.,^2019^). Glutamatergic interneurones apparently connect the T‐stellate cells and retrograde NO messaging at either of the input/output synapses of the interneurones can potentiate the release of glutamate and increase the gain applied to auditory signals passing through the T‐stellates. Conversely, if glycinergic neurones with local axons were also subject to NO based facilitation then they could cause a decrease in the gain of their local targets. NO can exert different effects depending on whether it interacts with neurotransmitter specific receptor complexes or voltage‐dependent ionic channels and its role within the brain appears to be very complex.

An additional factor in this complexity may be that there are micro‐compartments in the VCN that have very different ambient levels of NO. There is a wide range of staining intensity for nNOS in individual neurones of the VCN, but there is also a range of densities of nNOS containing neurones with some densely packed and some relatively isolated (Coomber et al., 2015). The mobility of NO and its action as a volume transmitter (Artinian et al., 2010) allows the regulation of neurones even if they do not generate NO themselves (Steinert et al., 2008). Some of this ambient NO may be produced by endothelial cells (Garthwaite, 2008). However, while the NO can freely diffuse in brain slices in vitro*,* the major mechanism for inactivating NO in vivo is the haemoglobin that is being pumped through a dense network of blood vessels in the VCN (Figure 2e, Santos et al., 2012). This may mean that the blood vessels form a meshwork that produces relatively isolated compartments. As a result, some neurones may be exposed to very low levels of NO even if they are very active, while others may be exposed to high levels of NO even if they are relatively inactive.

A further complication is the presence of the urethane anaesthetic, which is liable to affect the function of NO directly. The effects of anaesthesia are more of a concern at the higher levels of the auditory system and we have used chronic implants in our guinea pigs to record evoked potentials at the level of the auditory cortex and brainstem in awake guinea pigs (Berger, Coomber, Wallace, & Palmer, 2017) and compared them with anaesthetized. Anaesthesia does have an effect on the cochlear nucleus but it is more pronounced in the dorsal part. Activity in the VCN is affected to a lesser degree and the level of activity is remarkably resistant to anaesthesia (Evans & Nelson, 1973). However, there will be some effects partly due to descending inputs from the olivocochlear pathway. Very few recordings are made from the VCN in awake animals because it is difficult to gain access to it in a chronic preparation. It is not possible to be sure what effect the anaesthetic will have on the NO‐related drugs. Our main interest in the results is to compare them with results from animals that have developed tinnitus. A comparative study of that sort is less affected by anaesthesia than one designed to study the interactions between neurones in an alert animal.

Another potential confounding factor is the presence of different levels of reproductive hormones depending on the sex of the animal. There is some evidence that prolonged exposure to oestrogen and progesterone may influence the levels of nNOS in cortical neurones (Mannella, Sanchez, Giretti, Genazzani, & Simoncini, 2009). With all of our drugs, except SNOG, we used a mixture of male and female animals and overall sex should not have had much effect on our conclusions. With the SNOG results, all the animals were male and so fluctuating female hormone levels would not have been an issue. We were not aware of any effects in our results that might have been caused by differences in sex but the small numbers of neurones showing any particular effect means that we cannot rule it out entirely.

In the VCN, iontophoretic L‐NAME application (reduced NO levels) produced either increased or decreased spontaneous rates in about equal proportions. However, the tendency for L‐NAME to reduce high spontaneous rates and increase low spontaneous rates (Figure 8) suggest that the presence of NO is not acting to bring firing levels towards a long term mean value for a whole population, but rather is maintaining the range of spontaneous rates within the population. In other systems, NO has been shown to either increase or decrease spontaneous rates (Clasadonte, Poulain, Beauvillain, & Prevot, 2008; Kim et al., 2004; Podda et al., 2004), so the variable effect observed here is not unexpected.

In conclusion, our results show that some 16% of VCN neurones are under tonic suppression of their firing rate, while responding to acoustic stimulation, due to sustained effects of endogenous NO. The ambient levels of NO are also able to either facilitate or suppress the background firing rate, but this seems to affect less than 10% of neurones. However, other neurones (about 20%) show facilitation of their driven firing when exogenous NO is applied and potentially these neurones could be affected by a change in ambient NO levels that is thought to occur after noise exposure or the development of tinnitus (Coomber et al., 2014, 2015). Our previous work indicated that nNOS levels may increase in the VCN on the side that is exposed to high levels of unilateral noise while decreasing on the non‐exposed side. The functional effects may be complicated and we intend to study them directly in future in animals that have been noise‐exposed and then have gone on to develop tinnitus.

## CONFLICT OF INTEREST

The authors declare that the research was conducted in the absence of any commercial or financial relationships that could be construed as a potential conflict of interest.

## Data Availability

The original data files are available by contacting the first or last authors.

## References

[ejn14572-bib-0001] Ahern, G. P. , Klyachko, V. A. , & Jackson, M. B. (2002). cGMP and S‐nitrosylation: Two routes for modulation of neuronal excitability by NO. Trends in Neurosciences, 25, 510–517. 10.1016/S0166-2236(02)02254-3 12220879

[ejn14572-bib-0002] Alvarado, J. C. , Fuentes‐Santamaría, V. , Gabaldón‐Ull, M. C. , Jareño‐Flores, T. , Miller, J. M. , & Juiz, J. M. (2016). Noise‐induced “Toughening” effect in wistar rats: Enhanced auditory brainstem responses are related to calretinin and nitric oxide synthase upregulation. Frontiers in Neuroanatomy, 10, 1–24. 27065815 10.3389/fnana.2016.00019 PMC4815363

[ejn14572-bib-0003] Arnott, R. H. , Wallace, M. N. , Shackleton, T. M. , & Palmer, A. R. (2004). Onset neurons in the anteroventral cochlear nucleus project to the dorsal cochlear nucleus. Journal of the Association for Research in Otolaryngology, 5, 1153–1170. 10.1007/s10162-003-4036-8 PMC2538402 15357418

[ejn14572-bib-0004] Artinian, L. , Tornieri, K. , Zhong, L. , Baro, D. , & Rehder, V. (2010). Nitric oxide acts as a volume transmitter to modulate electrical properties of spontaneously firing neurons via apamin‐sensitive potassium channels. Journal of Neuroscience, 30, 1699–1711. 10.1523/JNEUROSCI.4511-09.2010 20130179 PMC6633990

[ejn14572-bib-0005] Auerbach, B. D. , Rodrigues, P. V. , Salvi, R. J. , Zhang, J. , Shaikh, A. G. , & Liu, B. (2014). Central gain control in tinnitus and hyperacusis. Frontiers in Neurology, 5, 1–21. 25386157 10.3389/fneur.2014.00206 PMC4208401

[ejn14572-bib-0006] Baizer, J. S. , Wong, K. M. , Paolone, N. A. , Weinstock, N. , Salvi, R. J. , Manohar, S. , … Hof, P. R. (2014). Laminar and neurochemical organisation of the dorsal cochlear nucleus of the human, monkey, cat, and rodents. Anatomical Record (Hoboken), 297, 1865–1884. 10.1002/ar.23000 PMC4170232 25132345

[ejn14572-bib-0007] Berger, J. I. , Coomber, B. , Wallace, M. N. , & Palmer, A. R. (2017). Reductions in cortical alpha activity, enhancements in neural responses and impaired gap detection caused by sodium salicylate in awake guinea pigs. European Journal of Neuroscience, 45, 398–409. 10.1111/ejn.13474 27862478 PMC5763375

[ejn14572-bib-0008] Brenman, J. E. , Xia, H. , Chao, D. S. , Black, S. M. , & Bredt, D. S. (1997). Regulation of neuronal nitric oxide synthase through alternative transcripts. Developmental Neuroscience, 19, 224–231. 10.1159/000111211 9208206

[ejn14572-bib-0009] Bullock, D. C. , Palmer, A. R. , & Rees, A. (1988). Compact and easy‐to‐use tungsten‐in‐glass microelectrode manufacturing workstation. Medical and Biological Engineering & Computing, 26, 669–672. 10.1007/BF02447511 3256764

[ejn14572-bib-0010] Burette, A. , Petrusz, P. , Schmidt, H. H. , & Weinberg, R. J. (2001). Immunohistochemical localization of nitric oxide synthase and soluble guanylyl cyclase in the ventral cochlear nucleus of the rat. The Journal of Comparative Neurology, 431, 1–10. 10.1002/(ISSN)1096-9861 11169986

[ejn14572-bib-0011] Cao, X.‐J. , Lin, L. , Sugden, A. U. , Connors, B. W. , & Oertel, D. (2019). Nitric oxide‐mediated plasticity of interconnections between T‐stellate cells of the ventral cochlear nucleus generate positive feedback and constitute a central gain control in the auditory system. Journal of Neuroscience, 39, 6095–6107. 10.1523/JNEUROSCI.0177-19.2019 31160538 PMC6668202

[ejn14572-bib-0012] Cao, X.‐J. , Shatadal, S. , & Oertel, D. (2007). Voltage‐sensitive conductances of bushy cells of the mammalian ventral cochlear nucleus. Journal of Neurophysiology, 97, 3961–3975. 10.1152/jn.00052.2007 17428908

[ejn14572-bib-0013] Carletti, F. , Ferraro, G. , Rizzo, V. , Friscia, S. , & Sardo, P. (2012). Modulation of in vivo GABA‐evoked responses by nitric oxide‐active compounds in the globus pallidus of rat. Journal of Neural Transmission, 119, 911–921. 10.1007/s00702-011-0760-0 22258796

[ejn14572-bib-0014] Chen, T. J. , Huang, C. W. , Wang, D. C. , & Chen, S. S. (2004). Co‐induction of growth‐associated protein GAP‐43 and neuronal nitric oxide synthase in the cochlear nucleus following cochleotomy. Experimental Brain Research, 158, 151–162. 15148562 10.1007/s00221-004-1886-1

[ejn14572-bib-0015] Chen, S.‐R. , Jin, X.‐G. , & Pan, H.‐L. (2017). Endogenous nitric oxide inhibits spinal NMDA receptor activity and pain hypersensitivity induced by nerve injury. Neuropharmacology, 125, 156–165. 10.1016/j.neuropharm.2017.07.023 28754372 PMC5585059

[ejn14572-bib-0016] Clasadonte, J. , Poulain, P. , Beauvillain, J. C. , & Prevot, V. (2008). Activation of neuronal nitric oxide release inhibits spontaneous firing in adult gonadotropin‐releasing hormone neurons: A possible local synchronizing signal. Endocrinology, 149(2), 587–596. 10.1210/en.2007-1260 18006627

[ejn14572-bib-0017] Collingridge, G. L. , & Lester, R. A. J. (1989). Excitatory amino acid receptors in the vertebrate central nervous system. Pharmacological Reviews, 41, 143–210. 2558391

[ejn14572-bib-0018] Coomber, B. , Berger, J. I. , Kowalkowski, V. L. , Shackleton, T. M. , Palmer, A. R. , & Wallace, M. N. (2014). Neural changes accompanying tinnitus following unilateral acoustic trauma in the guinea pig. European Journal of Neuroscience, 40, 2427–2441. 10.1111/ejn.12580 24702651 PMC4215599

[ejn14572-bib-0019] Coomber, B. , Edwards, D. , Jones, S. J. , Shackleton, T. M. , Goldschmidt, J. , Wallace, M. N. , & Palmer, A. R. (2011). Cortical inactivation by cooling in small animals. Frontiers in Systems Neuroscience, 5, 1–10. 21734869 10.3389/fnsys.2011.00053 PMC3122068

[ejn14572-bib-0020] Coomber, B. , Kowalkowski, V. L. , Berger, J. I. , Palmer, A. R. , & Wallace, M. N. (2015). Modulating central gain in tinnitus: Changes in nitric oxide synthase in the ventral cochlear nucleus. Frontiers in Neurology, 6, 1–12. 25806021 10.3389/fneur.2015.00053 PMC4354362

[ejn14572-bib-0021] Coote, E. J. , & Rees, A. (2008). The distribution of nitric oxide synthase in the inferior colliculus of guinea pig. Neuroscience, 154, 218–225. 10.1016/j.neuroscience.2008.02.030 18400412

[ejn14572-bib-0022] Doucet, J. R. , Ross, A. T. , Gillespie, M. B. , & Ryugo, D. K. (1999). Glycine immunoreactivity of multipolar neurons in the ventral cochlear nucleus which project to the dorsal cochlear nucleus. Journal of Comparative Neurology, 408, 515–531. 10.1002/(ISSN)1096-9861 10340502

[ejn14572-bib-0023] Eliasson, M. J. , Blackshaw, S. , Schell, M. J. , & Snyder, S. H. (1997). Neuronal nitric oxide synthase alternatively spliced forms: Prominent functional localizations in the brain. Proceedings of the National Academy of Sciences of the United States of America, 94, 3396–3401. 10.1073/pnas.94.7.3396 9096405 PMC20381

[ejn14572-bib-0024] Evans, E. F. , & Nelson, P. G. (1973). Responses of single neurons in cochlear nucleus of cat as a function of their location and anesthetic state. Experimental Brain Research, 17, 402–427. 4725899 10.1007/BF00234103

[ejn14572-bib-0025] Feelisch, M. , Ostrowski, J. , & Noack, E. (1989). On the mechanism of NO release from sydnonimines. Journal of Cardiovascular Pharmacology, 14(Suppl. 11), 13–22. 10.1097/00005344-198914110-00004 2484692

[ejn14572-bib-0026] Fessenden, J. D. , Altschuler, R. A. , Seasholtz, A. F. , & Schacht, J. (1999). Nitric oxide/cyclic guanosine monophosphate pathway in the peripheral and central auditory system of the rat. Journal of Comparative Neurology, 404, 52–63. 10.1002/(ISSN)1096-9861 9886024

[ejn14572-bib-0027] Fessenden, J. D. , Coling, D. E. , & Schacht, J. (1994). Detection and characterization of nitric oxide synthase in the mammalian cochlea. Brain Research, 668, 9–15. 10.1016/0006-8993(94)90505-3 7535658

[ejn14572-bib-0028] Friedland, D. R. , Eernisse, R. , & Popper, P. (2007). Potassium channel gene expression in the rat cochlear nucleus. Hearing Research, 228, 31–43. 10.1016/j.heares.2007.01.024 17346910 PMC1995076

[ejn14572-bib-0029] Galati, S. , D'Angelo, V. , Scarnati, E. , Stanzione, P. , Martorana, A. , Procopio, T. , … Stefani, A. (2008). In vivo electrophysiology of dopamine‐denervated striatum: Focus on the nitric oxide/cGMP signaling pathway. Synapse (New York, N. Y.), 62, 409–420. 10.1002/(ISSN)1098-2396 18361439

[ejn14572-bib-0030] Garthwaite, J. (2008). Concepts of neural nitric oxide‐mediated transmission. European Journal of Neuroscience, 27, 2783–2802. 10.1111/j.1460-9568.2008.06285.x 18588525 PMC2610389

[ejn14572-bib-0031] Garthwaite, J. (2019). NO as a multimodal transmitter in the brain: Discovery and current status. British Journal of Pharmacology, 176, 197–211. 10.1111/bph.14532 30399649 PMC6295412

[ejn14572-bib-0032] Goldberg, J. M. , & Brown, P. B. (1969). Response of binaural neurons of dog superior olivary complex to dichotic tonal stimuli: Some physiological mechanisms of sound localization. American Physiological Society, 32, 613–636. 10.1152/jn.1969.32.4.613 5810617

[ejn14572-bib-0033] Goldschmidt, J. , Wanger, T. , Engelhorn, A. , Friedrich, H. , Happel, M. , Ilango, A. , … Scheich, H. (2010). High‐resolution mapping of neuronal activity using the lipophilic thallium chelate complex TlDDC: Protocol and validation of the method. NeuroImage, 49, 303–315. 10.1016/j.neuroimage.2009.08.012 19682585

[ejn14572-bib-0034] Kim, H. W. , Park, J.‐S. , Jeong, H.‐S. , Jang, M. J. , Kim, B.‐C. , Kim, M.‐K. , … Park, S. W. (2004). Nitric oxide modulation of the spontaneous firing of rat medial vestibular nuclear neurons. Journal of Pharmacological Sciences, 96, 224–228. 10.1254/jphs.SCJ04006X 15492461

[ejn14572-bib-0035] Koerber, K. C. , Pfeiffer, R. R. , Warr, W. B. , & Kiang, N. Y. S. (1966). Spontaneous spike discharges from single units in the cochlear nucleus after destruction of the cochlea. Experimental Neurology, 16, 119–130. 10.1016/0014-4886(66)90091-4 5922930

[ejn14572-bib-0036] Kopp‐Scheinpflug, C. , Pigott, B. M. , & Forsythe, I. D. (2015). Nitric oxide selectively suppresses IH currents mediated by HCN1‐containing channels. Journal of Physiology, 593, 1685–1700. 10.1113/jphysiol.2014.282194 25605440 PMC4386966

[ejn14572-bib-0037] Lee, M. A. , Cai, L. , Hubner, N. , Lee, Y. A. , & Lindpaintner, K. (1997). Tissue‐ and development‐specific expression of multiple alternatively spliced transcripts of rat neuronal nitric oxide synthase. Journal of Clinical Investestigation, 100, 1507–1512. 10.1172/JCI119673 PMC508331 9294118

[ejn14572-bib-0038] Lee, J.‐J. , Cho, Y.‐W. , Huh, Y. , Cha, C. I. , & Yeo, S. G. (2008). Effect of nitric oxide on auditory cortical neurons of aged rats. Neuroscience Letters, 447, 37–41. 10.1016/j.neulet.2008.09.074 18840505

[ejn14572-bib-0039] Mannella, P. , Sanchez, A. M. , Giretti, M. S. , Genazzani, A. R. , & Simoncini, T. (2009). Oestrogen and progestins differently prevent glutamate toxicity in cortical neurons depending on prior hormonal exposure via the induction of neural nitric oxide synthase. Steroids, 74, 650–656. 10.1016/j.steroids.2009.02.011 19463685

[ejn14572-bib-0040] Manzoni, O. , Prezeau, L. , Marin, P. , Desagher, S. , Bockaert, J. , & Fagni, L. (1992). Nitric oxide‐induced blockade of NMDA receptors. Neuron, 8, 653–662. 10.1016/0896-6273(92)90087-T 1314618

[ejn14572-bib-0041] Ngodup, T. , & Trussell, L. O. (2019). Discovery of a novel inhibitory cell type in the cochlear nucleus. ARO Abstracts, 42, PS14 Available at https://www.aro.org/assets/docs/2019_aro_mwm_abstracts_final.pdf

[ejn14572-bib-0042] Noreña, A. J. (2011). An integrative model of tinnitus based on a central gain controlling neural sensitivity. Neuroscience and Biobehavioral Reviews, 35, 1089–1109. 10.1016/j.neubiorev.2010.11.003 21094182

[ejn14572-bib-0043] Oertel, D. , Wright, S. , Cao, X. J. , Ferragamo, M. , & Bal, R. (2011). The multiple functions of T stellate/multipolar/chopper cells in the ventral cochlear nucleus. Hearing Research, 276, 61–69. 10.1016/j.heares.2010.10.018 21056098 PMC3078527

[ejn14572-bib-0044] Oertel, D. , Wu, S. H. , Garb, M. W. , & Dizack, C. (1990). Morphology and physiology of cells in slice preparations of the posteroventral cochlear nucleus of mice. Journal of Comparative Neurology, 295, 136–154. 10.1002/(ISSN)1096-9861 2341631

[ejn14572-bib-0045] Olthof‐Bakker, B. M. J. , Gartside, S. E. , & Rees, A. (2019). Puncta of neuronal nitric oxide synthase (nNOS) mediate NMDA‐recptor signalling in the auditory midbrain. Journal of Neuroscience, 39, 876–887. 10.1523/JNEUROSCI.1918-18.2018 30530507 PMC6382984

[ejn14572-bib-0046] Palmer, A. R. , & Shackleton, T. M. (2009). Variation in the phase of response to low‐frequency pure tones in the guinea pig auditory nerve as functions of stimulus level and frequency. Journal of the Association for Research in Otolaryngology, 10, 233–250. 10.1007/s10162-008-0151-x 19093151 PMC2674197

[ejn14572-bib-0047] Palmer, A. R. , Wallace, M. N. , Arnott, R. H. , & Shackleton, T. M. (2003). Morphology of physiologically characterised ventral cochlear nucleus stellate cells. Experimental Brain Research, 153, 418–426. 10.1007/s00221-003-1602-6 12955380

[ejn14572-bib-0048] Perney, T. M. , & Kaczmarek, L. K. (1997). Localization of a high threshold potassium channel in the rat cochlear nucleus. Journal of Comparative Neurology, 386, 178–202. 10.1002/(ISSN)1096-9861 9295146

[ejn14572-bib-0049] Podda, M. V. , Marcocci, M. E. , Oggiano, L. , D'Ascenzo, M. , Tolu, E. , Palamara, A. T. , … Grassi, C. (2004). Nitric oxide increases the spontaneous firing rate of rat medial vestibular nucleus neurons in vitro via a cyclic GMP‐mediated PKG‐independent mechanism. European Journal of Neuroscience, 20, 2124–2132. 10.1111/j.1460-9568.2004.03674.x 15450091

[ejn14572-bib-0050] Rapisarda, C. , & Bacchelli, B. (1977). The brain of the guinea pig in stereotaxic coordinates. Archives of Science and Biology (Bologna), 61, 1–37. 400095

[ejn14572-bib-0051] Rhode, W. S. , Oertel, D. , & Smith, P. H. (1983). Physiological response properties of cells labelled intracellularly with horseradish peroxidase in cat ventral cochlear nucleus. Journal of Comparative Neurology, 213, 448–463. 10.1002/(ISSN)1096-9861 6300200

[ejn14572-bib-0052] Rodrigo, J. , Springall, D. R. , Uttenthal, O. , Bentura, M. L. , Abadia‐Molina, F. , Riveros‐Moreno, V. , … Moncada, S. (1994). Localization of nitric oxide synthase in the adult rat brain. Philosophical Transactions of the Royal Society of London, 345, 175–221. 7526408 10.1098/rstb.1994.0096

[ejn14572-bib-0053] Rothman, J. S. , & Manis, P. B. (2003). The roles potassium currents play in regulating the electrical activity of ventral cochlear nucleus neurons. Journal of Neurophysiology, 89, 3097–3113. 10.1152/jn.00127.2002 12783953

[ejn14572-bib-0054] Rusznák, Z. , Bakondi, G. , Pocsai, K. , Pór, A. , Kosztka, L. , Pál, B. , … Szucs, G. (2008). Voltage‐gated potassium channel (Kv) subunits expressed in the rat cochlear nucleus. Journal of Histochemistry and Cytochemistry, 56, 443–465. 10.1369/jhc.2008.950303 18256021 PMC2324191

[ejn14572-bib-0055] Santos, R. M. , Lourenço, C. F. , Ledo, A. , Barbosa, R. M. , & Laranjinha, J. (2012). Nitric oxide inactivation mechanisms in the brain: Role in bioenergetics and neurodegeneration. International Journal of Cell Biology, 391914 10.1155/2012/391914 22719764 PMC3376480

[ejn14572-bib-0056] Schaette, R. , & McAlpine, D. (2011). Tinnitus with a normal audiogram: Physiological evidence for hidden hearing loss and computational model. Journal of Neuroscience, 31, 13452–13457. 10.1523/JNEUROSCI.2156-11.2011 21940438 PMC6623281

[ejn14572-bib-0057] Schrammel, A. , Pfeiffer, S. , Schmidt, K. , Koesling, D. , & Mayer, B. (1998). Activation of soluble guanylyl cyclase by the nitrovasodilator 3‐morpholinosydnonimine involves formation of S‐nitrosoglutathione. Molecular Pharmacology, 54, 207–212. 10.1124/mol.54.1.207 9658207

[ejn14572-bib-0058] Smith, P. H. , & Rhode, W. S. (1987). Characterization of HRP‐labeled globular bushy cells in the cat anteroventral cochlear nucleus. Journal of Comparative Neurology, 266, 360–375. 10.1002/(ISSN)1096-9861 3693616

[ejn14572-bib-0059] Smith, P. H. , & Rhode, W. S. (1989). Structural and functional properties distinguish two types of multipolar cells in the ventral cochlear nucleus. Journal of Compatative Neurology, 282, 595–616. 10.1002/(ISSN)1096-9861 2723154

[ejn14572-bib-0060] Steinert, J. R. , Kopp‐Scheinpflug, C. , Baker, C. , Challiss, R. A. J. , Mistry, R. , Haustein, M. D. , … Forsythe, I. D. (2008). Nitric oxide is a volume transmitter regulating postsynaptic excitability at a glutamatergic synapse. Neuron, 60, 642–656. 10.1016/j.neuron.2008.08.025 19038221

[ejn14572-bib-0061] Steinert, J. R. , Robinson, S. W. , Tong, H. , Haustein, M. D. , Kopp‐Scheinpflug, C. , & Forsythe, I. D. (2011). Nitric oxide is an activity‐dependent regulator of target neuron intrinsic excitability. Neuron, 71, 291–305. 10.1016/j.neuron.2011.05.037 21791288 PMC3245892

[ejn14572-bib-0062] Tsuji, J. , & Liberman, M. C. (1997). Intracellular labeling of auditory nerve fibers in guinea pig: Central and peripheral projections. The Journal of Comparative Neurology, 381, 188–202. 10.1002/(ISSN)1096-9861 9130668

[ejn14572-bib-0063] Wallace, M. N. , & Bisland, S. K. (1994). NADPH‐diaphorase activity in activated astrocytes represents inducible nitric oxide synthase. Neuroscience, 59, 905–919. 10.1016/0306-4522(94)90294-1 7520136

[ejn14572-bib-0064] Yassin, L. , Radtke‐Schuller, S. , Asraf, H. , Grothe, B. , Hershfinkel, M. , Forsythe, I. D. , & Kopp‐Scheinpflug, C. (2014). Nitric oxide signaling modulates synaptic inhibition in the superior paraolivary nucleus (SPN) via cGMP‐dependent suppression of KCC2. Frontiers in Neural Circuits, 8, 65 10.3389/fncir.2014.00065 24987336 PMC4060731

[ejn14572-bib-0065] Zdanski, C. J. , Prazma, J. , Petrusz, P. , Grossman, G. , Raynor, E. , Smith, T. L. , & Pillsbury, H. C. (1994). Nitric oxide synthase is an active enzyme in the spiral ganglion cells of the rat cochlea. Hearing Research, 79, 39–47. 10.1016/0378-5955(94)90125-2 7528738

